# Metabolic complexity and nutritional reductionism. Deconstructing the single active ingredient paradigm in protein and amino acid supplements

**DOI:** 10.3389/fnut.2026.1970280

**Published:** 2026-10-05

**Authors:** Giovanni Corsetti, Evasio Pasini

**Affiliations:** 1 Department of Clinical and Experimental Sciences, Università di Brescia, Brescia, Italy; 2 Institute of Human Health, Ruaha Catholic University, Iringa, Tanzania

**Keywords:** amino acid supplements, dietary supplements, metabolic complexity, metabolism, protein supplements, reductionism, systems biology

## Abstract

Metabolism is an adaptive and highly interconnected system in which biological outcomes emerge from dynamic interactions among genes, proteins, metabolites, signaling pathways, microbiota, and environmental factors. Nevertheless, nutritional research has traditionally relied on a reductionist paradigm, attributing biological effects to isolated compounds acting on specific molecular targets through linear cause–effect relationships. While this approach has been instrumental in elucidating biochemical mechanisms and addressing nutrient deficiencies, its explanatory and predictive power is limited in complex biological systems and multi-component nutritional interventions. Dietary supplements, particularly protein- and amino acid-based formulations, may exert effects through synergistic or antagonistic interactions, network modulation, and context-dependent responses that cannot be adequately predicted from isolated molecular effects. Interindividual variability, metabolic plasticity, and feedback regulation further challenge deterministic models and may contribute to inconsistent clinical findings. A systems-based perspective complements reductionist approaches by considering metabolism as a dynamic network in which biological responses emerge from nonlinear interactions across multiple organizational levels. This narrative review specifically covers protein and amino acid supplement categories, including intact protein concentrates (whey, casein, collagen, soy, pea and rice), individual amino acids (Leucine, β-hydroxy-β-methyl butyrate), and amino acid mixtures (branched chain amino acids and essential amino acids mixture), to examine their role in metabolic health. Indeed, their widespread use is often supported by mechanistic evidence that may exceed available clinical data. Integrating mechanistic knowledge with systems biology may provide a more realistic framework for interpreting supplement efficacy, understanding biological variability, and improving the translational relevance of nutritional research and clinical practice.

## 1 Introduction

### 1.1 From reductionism to systems complexity: rethinking dietary supplements in metabolic health

Metabolism cannot be understood as the simple sum of isolated biochemical reactions. Rather, biological functions emerge from dynamic interactions among interconnected pathways, regulatory mechanisms, and environmental factors that collectively maintain homeostasis and influence disease development. This complexity poses a particular challenge to the study of dietary supplements, whose biological effects may depend not only on the properties of individual compounds but also on their interactions, synergistic or antagonistic effects, and the physiological context in which they act.

The use of dietary supplements to support metabolic health has increased substantially in both clinical settings and the general population. However, their mechanisms of action and biological efficacy remain incompletely understood. Nutritional and supplement research has traditionally adopted a reductionist paradigm, focusing on individual nutrients or bioactive compounds and their interactions with specific molecular targets according to a largely linear cause–effect model (1–3). This approach has provided major methodological advantages, including experimental control, standardization, and mechanistic resolution, and has contributed substantially to the identification of biochemical pathways and the prevention and treatment of deficiency diseases such as scurvy and rickets (1, 4). Nevertheless, its explanatory power becomes increasingly limited when applied to complex mixtures or adaptive biological systems.

Human metabolism is a highly interconnected and dynamic network involving genes, proteins, metabolites, signaling pathways, microbiota, and environmental factors operating across multiple spatial and temporal scales (5, 6). Its functional outcomes emerge from non-linear interactions, feedback mechanisms, adaptation, and context dependence and therefore cannot always be predicted from the behavior of individual components (7, 8). Synergistic and antagonistic interactions, network effects, and interindividual variability may substantially modify the effects of a given compound and limit the extrapolation of findings obtained from isolated molecular or biochemical systems to real-world physiological conditions (7, 8).

The distinction between reductionism and complexity is therefore not merely methodological but also epistemological. Reductionism seeks to explain biological phenomena by decomposing systems into constituent elements and identifying deterministic relationships between individual components and outcomes (2, 3). In contrast, a systems-complexity perspective seeks to understand how integrated biological systems behave as wholes, emphasizing non-linear interactions, feedback, adaptability, context dependence, and variability (5, 7, 8). In this framework, biological plasticity and interindividual differences are not simply sources of experimental noise but intrinsic properties of living systems (7, 9).

This conceptual distinction is particularly relevant to dietary supplements. Many supplements contain phyto-complexes or multiple bioactive ingredients whose effects may depend on interactions among constituents rather than on a single active compound or molecular target (10). From a systems perspective, their metabolic effects may be shaped by synergistic or antagonistic interactions, network modulation, and individual physiological characteristics, including genetic background, microbiota composition, nutritional status, and baseline metabolic function (7). An exclusively reductionist approach may consequently overestimate effects attributable to isolated components under controlled experimental conditions or, conversely, fail to capture clinically relevant effects that emerge only at the system level (8). Such limitations may also contribute to the inconsistent or discordant findings frequently observed in clinical studies of dietary supplements (11).

Reductionism nevertheless remains indispensable for identifying molecular mechanisms and generating mechanistic hypotheses. Its integration within a complexity-informed, systems-based framework may provide a more realistic interpretation of supplement efficacy, better account for biological variability and adaptive responses, and enhance the translational relevance of nutritional research (12).

Against this background, the primary objective of this narrative review is to critically examine the application of reductionist vs. systems-based approaches to dietary supplement research in metabolic health. The secondary objective is to review current evidence on widely used protein and Amino Acid (AA) supplements, where mechanistic hypotheses often exceed clinical outcomes, and re-interpret their potential effects through a systems biology lens.

## 2 Methods

Although predefined search terms were used to guide the literature search, this review was not conducted according to PRISMA guidelines and should therefore not be considered a systematic review. Given its broad and integrative scope, studies were selected based on their scientific relevance to key topics related to protein and AA supplementation, including protein sources, absorption, signaling pathways, and metabolic effects.

Relevant scientific studies were primarily identified through searches of databases such as PubMed and Web of Science, with a focus on recent studies (generally published within the last 15 years), using combinations of the following keywords: “protein supplementation,” “amino acid supplementation,” “essential amino acids,” “senescence,” “sarcopenia,” “hypercatabolic states,” “physical activity,” and “intense training.” Boolean operators (“and,” “or”) were used to refine and expand the search strategy. Human and animal studies published in English through May 2026 were considered. Priority was given to mechanistic studies, influential preclinical evidence, peer-reviewed original clinical studies, and human studies providing translational insights into the physiological effects of protein and AA supplementation. Meta-analyses, systematic reviews, and narrative reviews were also considered. Studies reporting neutral or conflicting findings were also included to ensure a balanced representation of the available evidence. Additional references were included based on their relevance to the topic and conceptual contribution. Overall, this approach provided a thorough yet targeted synthesis of the available evidence on the effects of dietary supplements.

To improve the transparency of the reporting of this narrative review, we followed the guidelines proposed by the Standards for Reporting Qualitative Research (SRQR) (13). This framework provided a structured approach to enhance methodological rigor, minimize reporting bias, and ensure a transparent, consistent synthesis of the conceptual literature. Generative AI was used to create part of Figure 1 and to check the grammar.

**Figure 1:**
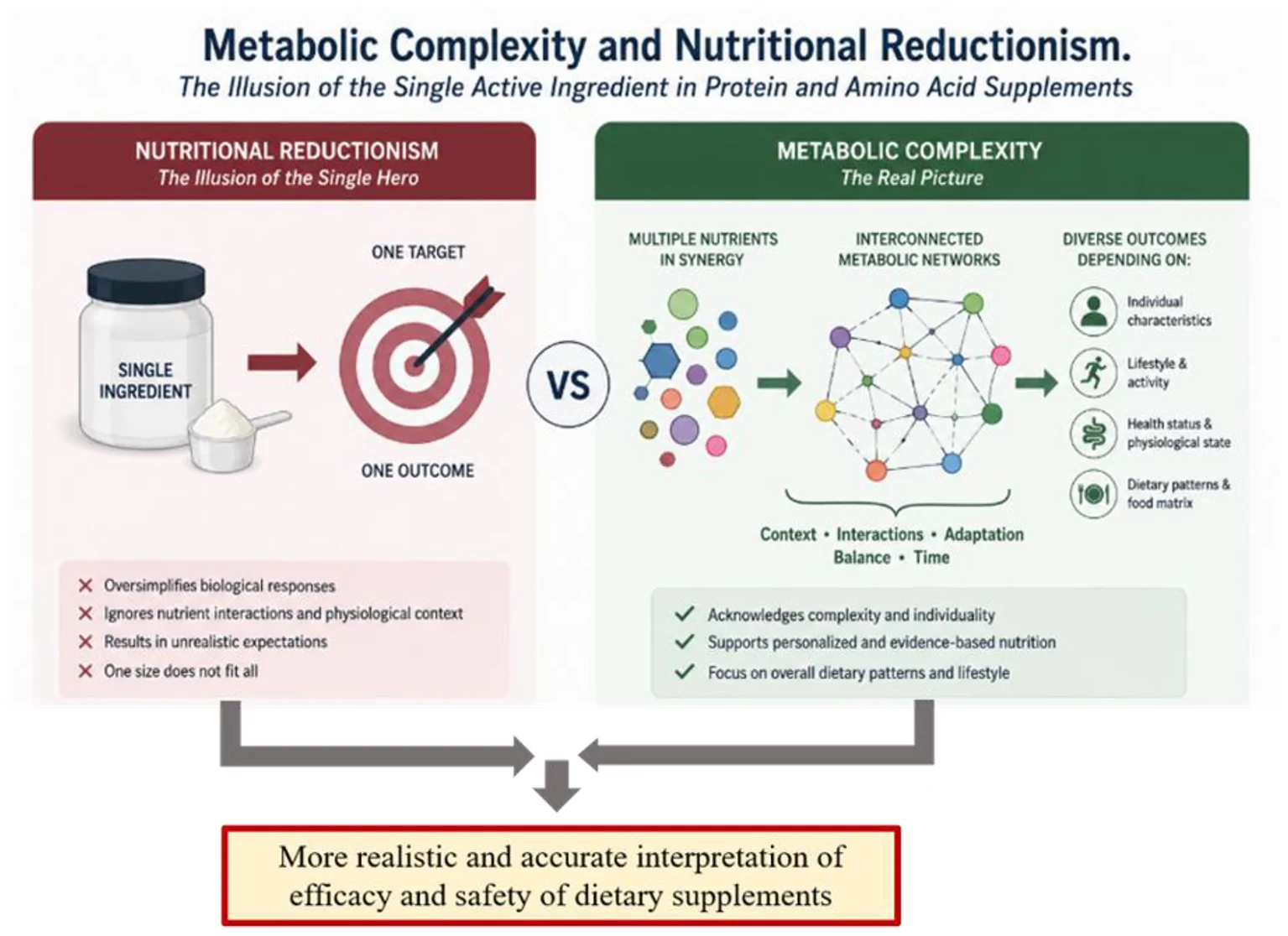
Schematic representation of the conceptual differences between the traditional reductionist approach and metabolic complexity. Although reductionism has been instrumental in elucidating specific biochemical mechanisms, metabolism functions as a dynamic and highly inter-connected network, whose responses emerge from the interactions of many factors. Integrating mechanistic knowledge with systems biology provides a more realistic framework for understanding the efficacy of dietary supplements and improving their implementation in nutritional and clinical practice.

## 3 The balanced diet paradigm: an integrative approach to protein and amino acid nutrition

The use of nutritional supplements, particularly protein- and Amino Acid (AA)-based products, has increased substantially over recent decades, driven in part by marketing strategies promoting broad and often nonspecific benefits for health, physical performance, and body composition. This widespread use raises a fundamental question: are such supplements genuinely necessary, or are they justified and effective only under specific physiological or pathological conditions?

A core principle of modern nutrition science is that a varied and balanced diet can meet the macro- and micronutrient requirements of healthy individuals. According to current European guidelines, the average daily protein requirement for healthy adults is approximately 0.83 g/kg body weight, an intake readily achievable through a conventional diet, which often provides protein more than minimal physiological requirements (14). In this context, routine protein supplementation does not generally address a physiological necessity but rather reflects individual preferences or beliefs that may not be supported by robust evidence.

Although dietary supplements are legally defined as “concentrated sources of nutrients intended to complement the normal diet,” they are often perceived as essential tools for optimizing health or enhancing physical performance (15). However, available evidence indicates that protein and AA supplementation provides limited additional benefit when energy and protein requirements are already adequately met through whole foods (16). Supplementation therefore becomes relevant when a gap arises between nutritional intake and metabolic requirements that cannot be adequately bridged through diet alone.

Specific physiological, clinical, and performance-related conditions may justify or require protein or AA supplementation. These situations are typically characterized by increased metabolic demands and/or a hypercatabolic state, in which endogenous protein turnover and nutrient requirements exceed what can be reasonably supported by conventional dietary intake alone (17).

### 3.1 Senescence and sarcopenia

In older adults, the onset of anabolic resistance requires a higher protein intake (1.0–1.2 g/kg/day) to effectively stimulate muscle protein synthesis (MPS). When loss of appetite or chewing difficulties prevents reaching these levels, the use of isolated proteins (e.g., whey protein) or EAA blends is clinically justified to counteract muscle loss (18, 19). Following supplementation, sarcopenic or malnourished subjects tend to show better favorable responses than well-nourished subjects (20–24). However, some recent meta-analyses highlight a high methodological heterogeneity, with various types of supplements (whey protein, EAA, leucine/BCAA, etc.,), dosages and duration (23) making it necessary to adopt great caution in generalizing the conclusions. What is certain, however, is that protein/AA supplementation is more effective in improving strength and physical performance when combined with resistance exercise (25) compared to supplementation alone or exercise alone (26, 27) (Table 1).

**Table 1:** Summary of some meta-analysis (MA) and randomized clinical trial (RCT) on effects of protein supplementation in senescence and sarcopenia.

References	Study	Population	Intervention	Main results
Xie et al. (26)	MA 9 studies	Sarcopenic adults ≥65. *n* = 496	RT vs RT + BCAA or EAA or HMB; different protocols.	↑ strength, gait speed and SPPB; no effect on LBM
Yoshimura et al. (27)	MA 13 studies	Sarcopenic or frail adults ≥65. *n* = 1,057	Protein supplementation, or combined with PA	Protein + PA: low ↑LBM and strength vs. PA alone. Very low quality of evidence.
Tu et al. (165)	MA 12 studies	Sarcopenic and frail elderly ≥65 *n* = 872	15 to 40 g/day protein or 3 g/day HMB. 2–12 months	No effect on physical performance, LBM and strength
Björkman et al. (166)	RCT	Sarcopenic elderly ≥74. *n* = 218	WP 40 g/d + low-PA/D3 vs. placebo. 12 months.	No improvement on LBM and strength
Ten Haaf et al. (24)	RCT	PA elderly 67–73 *n* = 116	31 g/day milk protein vs. placebo. 12 weeks.	↑ LBM, ↓ body fat. No improvement of strength
Rondanelli et al. (21)	RCT	Sarcopenic elderly. *n* = 159	EAA-mix 4g/day + melatonin 1mg/day vs. placebo. 4 weeks.	The intake of melatonin alone tends to worsen protein metabolism that can be counteracted by supplementation with EAA.
Cheng et al. (23)	MA 39 studies	Sarcopenic, frail or malnourished adults ≥65. *n* = 4,274	Protein or EAA-mix or single EAA supplements	Small benefits on LBM, strength, and function; high heterogeneity. EAA-mix may be most effective in undernourished subjects
Rondanelli et al. (20)	RCT	Sarcopenic elderly. *n* = 130	WP (22 g/d) + EAA (10.9 g/d) + Vit D + PA. 12 weeks.	↑ LBM, ↑ strength, ↑ physical function
Xu et al. (25)	MA 9 studies	Elderly ≥65. *n* = 511	Leucine or AA, or protein supplement vs. placebo. 10 days to 6 months. 10 days to 6 months.	No effect on LBM or strength

Arrow up, increase; Arrow down, decrease; RT, resistance training; WP, whey protein; EAA, essential amino acid; LBM, lean body mass; SPPB, Short Physical Performance Battery scores; PA, physical activity; BCAA, branched chain AA.

### 3.2 Clinical and para-physiological conditions

Protein-energy malnutrition, pregnancy and breastfeeding, clinical or para- physiological conditions, pathologies (oncological, respiratory failure, chronic inflammatory diseases, etc.,), malabsorption, bariatric surgery, post-surgical or post-traumatic periods, require specific nutritional protocols and/or targeted oral supplementation (28). Catabolism in critical conditions can lead to anabolic resistance, reducing the effectiveness of simple supplements (17). In such conditions, individualized nutritional supplementation is a fundamental therapeutic tool to be combined with standard pharmacological therapy. In these conditions, protein/AA supplementation has proven effective in improving nitrogen balance and body mass in short-term studies (29). Pre-clinical and metabolic logic supports the idea that single AA (e.g., arginine, glutamine) or mixes of all EAA can modulate the anabolic response in Hypercatabolic States (HCS) and inflammatory conditions (17, 30). However, some RCTs in critically ill patients do not demonstrate a clear beneficial effect of supplementation on muscle loss, strength, or muscle function (31, 32). Systematic reviews and meta-analyses highlight methodological heterogeneity and the lack of consistent evidence of efficacy, suggesting a low certainty of beneficial action (33, 34). Indeed, the diversity of interventions (type of protein and AA, doses, duration) makes it difficult to compare studies (34) (Table 2). Furthermore, in all studies, supplementation is always part of standard nutrition, so it is impossible to separate the specific effect of supplements from that of foods (35).

**Table 2:** Randomized clinical trials (RCTs) and systematic (S) or narrative (N) reviews (R) of on effects of protein supplementation in hypercatabolic states (HCS).

References	Study	Population	Intervention	Main results
Veldsman et al. (31)	RCT	ICU patients (37 ± 12y). *n* = 50	10–12g EAA + mobilization vs. standard care. 8 days.	No improvement in LBM and strength/function vs. control
Corsetti and Pasini, (17)	NR	Heterogeneous HCS conditions	AA/EAA/proteins under heterogeneous HCS	Theoretical support that targeted EAAs may help overcome anabolic resistance; more RCTs needed.
Wittholz, et al. (34)	MA 30 studies	ICU patients. *n* = 1,976	Enteral AA or metabolites vs. control	High heterogeneity; effects uncertain; some studies with HMB show attenuation of catabolism, but the evidence is weak.
Heming et al. (32)	RCT	ICU patients. *n* = 35	Enteral 5AA-blend (threonine, cysteine, proline, serine and leucine) vs. placebo. 21 days	Proof-of-concept study, no significant reduction in muscle degradation, but improved quadriceps, diaphragm and gut functions.
Ragni et al. (30)	NR	Murine and human tumor models	Cachexia prevention with AA (Arg, Gln, EAA) supplementation vs. control	Preclinical evidence that EAAs, Arg, and Gln can improve energy balance and counteract cachexia.
Wandrag et al. (33)	SR	4 ICU patients, *n* = 174 18 non-ICU *n* = 388	AA and metabolites vs. standard care	Heterogeneous evidence; conflicting results on LBM preservation during critical catabolism.
Weijs et al. (167)	NR 15 studies	ICU and malnourished patients. *n* = 12,408	Heterogeneous Protein/AA supplementation	Possible benefit of protein/AA support in ICU patients.
Bonnefoy et al. (29)	RCT	Malnourished and inflamed patients. *n* = 26	BCAA (0.4 and 0.2 g/kg/day) enriched protein vs. control. 2 weeks.	Favorable effects on nitrogen balance and body composition; increases protein synthesis in moderate catabolism.

MA, meta-analysis; ICU, in- tensive care unit; EAA, essential amino acids; BCAA, branched chain AA.

### 3.3 Elite athletes and intense training

In the context of sports, the use of protein supplements is often justified by supposed superior effects on muscle hypertrophy, strength, and recovery. However, current evidence suggests that, when total protein intake and protein dose are adequate, the source of protein (whole food vs. supplemental protein) has a relatively limited impact on long-term muscle adaptations, although protein quality, AA composition, and the distribution of protein intake across the day may influence the anabolic response (36). Supplementation appears more like a logistical convenience than a real biological necessity, especially in individuals who can plan their meals correctly. For athletes who undertake high training volumes, protein requirements can increase to 1.4–2.0 g/kg/day (37, 38). In situations such as travel and/or limited recovery windows between intense physical activity sessions, the administration of protein supplements offers a practical and rapidly assimilated advantage to optimize muscle recovery (39). Protein (especially whey) and EAA blends support increased post-exercise muscle protein synthesis and potentially improved recovery/damage biomarkers in elite athletes or during intense training, particularly when combined with carbohydrates or specific nutritional strategies (40, 41). Whey proteins in association with EAA have demonstrated superior efficacy to the use of proteins alone in improving net protein balance and in some cases muscle protein synthesis (42). Isolated BCAA shows limited and non-significant benefits on performance, muscle mass and strength, with some effects on the perception of post-exercise muscle soreness (43, 44). Many studies indicate that, if total daily protein intake is already adequate, adding isolated AA (e.g., BCAA) does not produce substantial improvements in performance or body composition above and beyond normal protein intake (45) (Table 3).

**Table 3:** Systematic reviews of RCTs on the effects of protein and/or AAs supplementation in elite athletes or those with high-volume training.

References	Study	Population	Intervention	Main results
Martinho et al. (43)	SR	Athletes of various sports and physically active people. *n* = 376	BCAA supplementation. Heterogeneous protocols.	BCAA supplementation does not have a significant impact on performance. Oral isolated BCAA reduces muscle soreness.
Master and Macedo (40)	SR	Athletes of various sports.	WP, BCAA, glutamine or leucine supplementation. Heterogeneous protocols.	Unlike proteins, BCAA, glutamine or leucine supplementation are equivocal and are not warranted.
Park et al. (42)	RCT	Young adult athletes. *n* = 16	EAA/WP supplement (6.3 g and 12.6 g), vs WP	Balanced EAA formulation combined with WP is highly anabolic as compared to a WP-based recovery product. The response is dose dependent.
Lam et al. (41)	MA 15 studies	Athletes of various sports. *n* = 230	Effect of WP, EAA, creatinine kinase and myoglobin. 2 to 60 days. Heterogeneous protocols.	Improvements in biomarkers of muscle damage and aminoacidemia, with potential benefits on recovery but high variability between studies. Evidence supports the effectiveness of WP as a positive ergogenic aid on athletes'AA, creatinine kinase and myoglobin.
Vieillevoye et al. (168)	RCT	Young males trained. *n* = 29	EAA + carbohydrates vs placebo during heavy training. 12 weeks.	EAA has a positive effect on muscle hypertrophy and architecture. Such a nutritional intervention seems to be more effective in subjects having lower nitrogen balance and/or lower initial strength.

SR, systematic review; MA, meta-analysis; RCT, randomized clinical trial; WP, whey protein; BCAAs, branched chain AAs.

The diversity of results across disciplines, dosages, time points, and populations, as well as the variability in experimental conditions and the lack of rigorous control of overall protein intake in the subjects studied, represent important limiting factors that reduce the strength of the conclusions.

Nutritional supplementation should therefore not be understood and considered a routine practice for the general population, but rather as a targeted and personalized nutritional assessment based on metabolic status (Table 4). Unfortunately, the gap between actual and perceived needs is largely fueled by marketing strategies that im-properly transfer evidence from specific populations to the average consumer.

**Table 4:** Summary of nutritional supplementation conditions based on physiological context and clinical target.

Physiological context	Main clinical target	Rationale for integration
Healthy adult subject (sedentary)	Maintaining homeostasis	Rarely necessary: if the diet is balanced it covers the entire protein/AA requirement.
Athletes (strength/endurance)	Tissue repair and hypertrophy	Useful for convenience and timing optimization in post-workout.
Elderly/sarcopenic	Counteracting protein catabolism and anabolic resistance	Necessary/recommended in case of reduced caloric/protein intake or senile anorexia.
ICU/bariatric/clinical patient	Prevention and/or control of protein hyper catabolism	Mandatory for structural deficiencies or malabsorption.

In conclusion, to contextualize the translational relevance of current research, the hierarchy and strength of evidence supporting protein and AA supplementation vary substantially across different target populations. In athletic and resistance-trained cohorts, evidence is predominantly driven by high-quality RCT and systematic reviews focused on performance and hypertrophic adaptations. In older adults and sarcopenic populations, robust clinical RCT data demonstrate efficacy in blunting anabolic resistance, although heterogeneity in baseline nutritional status often moderates clinical outcomes. Conversely, in general healthy populations and broader clinical cohorts, the evidence base leans more heavily on observational studies, nitrogen balance models, and short-term mechanistic markers, where translational clinical benefits remain less defined (Table 5).

**Table 5:** Hierarchy and quality of scientific evidence for protein and amino acid supplementation across different target populations.

Target population	Dominant level of evidence	Key measured outcomes	Main evidence limitations and gaps
**Athletes and resistance-trained**	**High** *(Multiple RCT, MA)*	Muscle hypertrophy, peak power, post-exercise recovery	Potential ceiling effects; overuse of surrogate performance metrics
**Older adults and sarcopenic**	**Moderate to high** *(Clinical RCT, MPS tracer studies)*	MPS, frailty reduction, functional mobility	Variable baseline protein intake; underlying chronic inflammation confounding
**Clinical and hospitalized**	**Moderate** *(Heterogeneous RCT, Observational)*	Nitrogen balance, length of hospital stays, clinical recovery	High pathology-driven interindividual variability; adherence challenges
**General healthy adults**	**Low to Moderate** *(Epidemiological, Mechanistic studies)*	Biomarkers, baseline nitrogen status, general health markers	Sparse long-term RCT data; limited benefit over whole-food protein sources

MA, meta-analysis; MPS, muscle protein synthesis; RCT, randomized clinical trial.

When, following a nutritional assessment, supplementation is deemed appropriate, the choice of protein sources or AAs blends should be based on available scientific evidence, biological quality, bioavailability, and product safety, rather than on accompanying commercial claims that always promise miraculous effects.

## 4 Protein quality: from amino acid composition to dietary context

Protein quality reflects the capacity of a dietary protein to provide adequate amounts of digestible EAA to support protein synthesis and physiological functions. Historically, protein quality was assessed using Biological Value (BV), defined as the proportion of absorbed nitrogen retained for tissue protein synthesis. Proteins with a high BV have an amino acid profile closely matching human requirements and high digestibility. Egg protein has traditionally been considered the reference protein (BV≈100), whereas whey proteins may exceed this value because of their high leucine content and rapid digestibility (46, 47).

Although BV remains useful for historical comparisons, it does not account for the digestibility of individual indispensable amino acids. The Protein Digestibility-Corrected Amino Acid Score (PDCAAS) was subsequently introduced and later superseded by the Digestible Indispensable Amino Acid Score (DIAAS), proposed by the Food and Agriculture Organization (FAO) (48). Unlike BV and PDCAAS, DIAAS considers the true ileal digestibility of individual EAAs, providing a more physiologically relevant estimate of protein quality and its ability to meet human amino acid requirements. Recent evidence supports DIAAS as the most appropriate current method for assessing dietary protein quality (49).

However, protein quality is a multidimensional property that extends beyond amino acid composition and digestibility. It depends on total EAA content; the relative proportion of EAA to human requirements; protein digestibility; post-digestive AA bioavailability; the presence of limiting EAA; and the food matrix and antinutritional factors that may affect digestion or absorption. Thus, protein quality should be considered not only a property of the protein itself but also of the whole food matrix and dietary context (50).

Animal-derived proteins, including eggs, milk, whey, meat, poultry, and fish, generally provide highly digestible EAA in proportions closely matching human requirements, with digestibility often exceeding 90–95%. Their DIAAS values frequently exceed 100, placing them among the highest-quality dietary proteins according to FAO criteria; whey, casein, milk, and egg proteins are particularly well characterized in this regard (49). By contrast, many plant proteins have lower amounts of one or more limiting EAAs and lower digestibility, partly because of fiber, phytates, tannins, and other antinutritional factors. Cereals are typically limited in lysine, whereas legumes tend to provide lower amounts of sulfur-containing EAA, particularly methionine and cysteine. Consequently, plant proteins often have lower DIAAS values, although substantial differences exist among sources; pea protein generally shows intermediate values, whereas rice protein tends to score lower (51, 52).

Nevertheless, the traditional classification of plant proteins as “incomplete proteins” is increasingly considered outdated. Most plant protein sources contain all EAA, although some are present in lower proportions than in animal proteins. Protein quality should therefore be evaluated within the context of the overall dietary pattern rather than individual foods. Consuming diverse plant protein sources throughout the day can provide complementary amino acid profiles and compensate for limiting EAA, without requiring simultaneous protein combining. For example, cereal–legume combinations such as rice and beans or wheat and lentils improve the overall balance of lysine and sulfur-containing EAA, bringing protein quality closer to that of high-quality animal proteins (52–54). Table 6 summarizes the comparison of the main differences between characteristics of animal and plant proteins.

**Table 6:** Summary comparison of the main differences between characteristics of animal and plant proteins.

Characteristic	Animal proteins	Plant proteins
DIAAS	Usually, >100	Variable, 40–100
EAAs profile	Complete	One or more EAAs may be present in lower proportions
Digestibility	High (>90–95%)	Lower (70–90%), depending on source and processing
Leucine content	High	Generally lower
Limiting AA	Rare	Common (Lysine in cereals; methionine/cysteine in legumes)
Effect on MPS	Higher	Comparable when protein dose and AA balance are optimized.
Complementation required	No	Often beneficial

MPS, muscle protein synthesis; DIAAS, Digestible Indispensable-AA-Score.

Human intervention studies further indicate that well-planned predominantly plant-based diets can support muscle protein synthesis, preserve lean body mass, and maintain physical performance when total energy and protein intake are adequate and attention is given to leucine content, EAA composition, and protein distribution across meals. Nevertheless, compared with whey and other dairy proteins, many plant proteins have lower leucine content, digestibility, and EAA density. Achieving comparable anabolic responses may therefore require higher total protein intake, complementary protein sources, or leucine/EAA fortification, particularly in older adults and other populations characterized by anabolic resistance (50, 55).

Accordingly, protein quality is increasingly viewed not as a simple classification of proteins as “high-” or “low-quality” based on BV, but as a multidimensional concept integrating EAA composition, digestibility, bioavailability, food matrix, and dietary context. This broader perspective is particularly relevant when evaluating sustainable dietary patterns, in which combinations of complementary plant proteins may provide adequate protein quality while potentially offering additional environmental and cardiometabolic advantages.

## 5 Protein supplements

### 5.1 Whey Protein (WP)

WP are among the most extensively studied and widely used protein supplements, particularly in the context of sports nutrition and metabolic health. WP accounts for approximately 20% of bovine milk protein (the remaining 80% is casein). They are derived from the liquid whey left after cheesemaking and then subjected to filtration and purification to concentrate the protein fraction. WP consist primarily of β-lactoglobulin (≈50–55%) and α-lactalbumin (≈20%), with smaller amounts of bovine serum albumin, immunoglobulins, lactoferrin, and glycomacropeptides. Their AA profile is rich in EAA, particularly leucine, which underpins the scientific interest in WP's effects on muscle protein synthesis and energy metabolism (56, 57).

WP intake can produce modest reductions in fat mass and increases in lean mass in healthy adults, especially when combined with resistance exercise. A meta-analysis of randomized controlled trials reported improvements in body composition markers (fat mass, lean mass) in participants supplementing with WP vs. controls (58). However, in older adults, the impact of WP on muscle strength and physical function appears limited unless paired with a structured resistance training program that drives neuromuscular adaptation and hypertrophy (59, 60).

In subjects with metabolic syndrome or related metabolic disturbances, some clinical studies suggest that WP may improve glycaemic control and lipid profiles, although the overall consistency of findings is limited (61). A recent meta-analysis reported that WP supplementation can reduce LDL-cholesterol and total cholesterol, particularly in overweight/obese adults under 50 years of age, with stronger effects when combined with physical exercise (62). Conversely, evidence from heterogeneous populations suggests only modest effects on lipid profiles overall, with significant reductions in triglycerides but not in HDL or LDL (63).

Regarding systemic inflammation, the available evidence, unfortunately heterogeneous and not fully consistent, does not support firm conclusions. Some analyses identify small reductions in C-reactive protein with higher WP doses and/or in the presence of chronic inflammation (64). Among oncology and malnourished patients, preliminary data indicate that protein supplementation may help maintain body weight or lean mass, but the paucity of randomized trials precludes definitive conclusions (65, 66).

Although WP are generally perceived as safe, some reviews based on observational studies or non-systematic reports have noted potential adverse effects associated with chronic, unsupervised use of protein supplements, including signals of altered liver and kidney function, as well as gastrointestinal disturbances and changes in the gut microbiota (67).

In conclusion, while many publications support a favorable role for WP in specific contexts (exercise, body composition, lipids), the observed clinical effects are often modest and highly context-dependent (life stage, level of physical activity, metabolic status), which contributes to inconsistent results across studies. Substantial variability in study protocols (dose, formulation, duration, background diet) further complicates generalization and cautions against uncritical extrapolation of findings.

Thus, the available evidence does not always match the broad health and performance claims promoted commercially, underscoring the gap between the context-dependent effects documented in clinical studies and the more generalized benefits often attributed to WP supplementation.

### 5.2 Casein

Casein represents approximately 80% of the protein fraction of bovine milk and is traditionally classified as a “slow-release” protein, as opposed to WP, which are considered “fast-release.” This distinction stems primarily from their different digestive kinetics: casein coagulates in the gastric environment, slowing stomach emptying and resulting in a more gradual release of AA into the bloodstream (68). Unlike WP, this results in prolonged aminoacidemia, associated with a reduction in body proteolysis (68). This different absorption kinetics has fuelled numerous commercial claims that casein is a “strongly anti-catabolic” protein, particularly recommended before bed to maintain an anabolic state throughout the night. In the 1990's, the distinction “casein = anti-catabolic” and “WP = anabolic” had a strong influence on both research and marketing. Today, this dichotomy has been debunked. In fact, although these claims have a physiological basis, scientific literature suggests that the true clinical impact of supplementation tends to be overestimated. Studies conducted using labeled AA infusions have confirmed that the evening ingestion of approximately 30–40 g of casein increases AA availability and stimulates MPS, especially when combined with strength training performed in the hours preceding sleep (69, 70). However, more recent systematic reviews highlight that these acute effects translate into only modest benefits on chronic adaptations and are strongly influenced by overall daily protein intake and the quality of training, rather than solely by the timing of supplementation. Furthermore, pre-sleep casein supplementation appears to have limited or no effects on energy metabolism, appetite, and lipid oxidation, both in healthy subjects and in overweight or obese individuals (71).

The comparison between casein and WP is probably the most studied aspect. In general, WP induces a faster anabolic response and a higher peak in muscle protein synthesis in the hours immediately following ingestion, primarily due to the higher leucine content (72, 73). In contrast, casein causes a less intense but more prolonged response (68). However, when considering clinically relevant endpoints, such as in-creases in LBM, muscle strength, or training adaptations after weeks or months of supplementation, the differences between casein and WP are minimal or absent, provided that total dietary protein intake is adequate (74). In fact, the total amount of protein consumed during the day is the main determinant of muscular adaptations (16), while the choice between casein and WP is of secondary importance in most physically active individuals (75).

In conditions of increased protein catabolism, the slow digestion of casein could theoretically promote a positive protein balance during prolonged periods of fasting; however, available evidence does not demonstrate a clear superiority of casein over other high-quality nutritional proteins. In elderly subjects, anabolic resistance makes the amount of protein ingested per meal and the leucine content particularly important. In this context, protein-rich protein, due to its faster digestion and higher leu-cine content, generally stimulates a greater acute MPS response than casein. However, there is no convincing evidence that this advantage translates into greater hypertrophy or superior long-term strength gains when overall protein intake is adequate (76–78).

Unfortunately, most preclinical studies are not aimed at demonstrating specific therapeutic effects of casein but use this protein as a control in purified diets (e.g., in AIN-76 and AIN-93 formulations), to compare new protein sources or different nutritional interventions (79). This limits the possibility of attributing specific biological properties to casein based on experimental data alone.

In conclusion, available scientific evidence suggests that casein is a high-quality protein source, effective in helping meet daily protein requirements and supporting muscle protein synthesis, especially when consumed before bedtime. Claims that it is significantly superior to WP or a powerful anti-catabolic agent are not fully supported by the clinical literature (80, 81). The observed differences mainly concern digestive physiology and acute effects on protein synthesis (71, 82). Under most physiological conditions, long-term adaptations depend predominantly on the total amount of high-nutrient protein, its daily distribution, the quality of the food and the training stimulus.

The findings indicate that the scientific evidence for casein does not always align with the broad anabolic and “anti-catabolic” claims promoted in commercial marketing, as its long-term effects appear to depend more on total protein intake, dietary distribution, and training stimulus than on its slower digestion *per se*.

### 5.3 Collagen

Oral hydrolysed collagen (HCol) supplementation is widely marketed to improve age-related changes in skin, connective tissues, and bone health. Collagen is the most abundant structural protein in the human body, comprising approximately 30% of total body protein and encompassing at least 28 genetically distinct types that provide mechanical support to the skin, tendons, ligaments, cartilage, and bone. Despite its structural importance, collagen is considered a low-quality dietary protein because it contains relatively low amounts of EAAs, particularly leucine, and lacks tryptophan, making it unsuitable as a protein source for stimulating MPS. The most consistent evidence concerns modest improvements in skin hydration and elasticity after 8–12 weeks of HCol supplementation (2.5–10 g/day). However, effects on wrinkle reduction are generally small and of uncertain clinical relevance (83).

Current evidence suggests that the potential benefits of HCol supplementation are largely tissue-specific rather than systemic and are primarily related to skin and connective tissue metabolism. Several systematic reviews and meta-analyses of RCTs have reported small-to-moderate improvements in skin hydration, elasticity, and wrinkle appearance following daily HCol supplementation for 8–24 weeks. Proposed mechanisms include the absorption of collagen-derived peptides together with AA precursors, which may stimulate dermal fibroblast activity, Extracellular Matrix (ECM) turnover, and collagen synthesis, although the precise biological pathways remain incompletely understood (84, 85).

Similarly, some clinical studies suggest modest improvements in bone mineral density or bone turnover markers, particularly in postmenopausal women, while evidence also indicates potential benefits for tendon and ligament remodeling when collagen supplementation is combined with appropriately timed mechanical loading or exercise. Nevertheless, these effects appear to be modest and highly dependent on the population studied, supplementation protocol, exercise stimulus, and duration of intervention (85, 86).

The overall certainty of the evidence remains low to moderate. Many available RCTs include relatively small sample sizes, short intervention periods, heterogeneous collagen formulations and dosages, and inconsistent assessment of participants' habitual dietary protein intake. Furthermore, several meta-analyses have highlighted that a substantial proportion of positive findings originate from industry-funded studies, whereas independent trials generally report smaller or non-significant effects, raising concerns regarding publication and sponsorship bias (83, 87, 88).

From a physiological perspective, orally ingested HCol is not selectively incorporated into the skin or connective tissues. Like all dietary proteins, it undergoes gastro-intestinal digestion into free AA and small peptides before absorption. These metabolites enter the systemic AA pool and are subsequently utilized according to whole-body metabolic demands rather than being preferentially delivered to colla-gen-rich tissues (84). Consequently, endogenous collagen synthesis primarily depends on the overall availability of AA precursors, adequate vitamin C status, and the anabolic stimulus provided by mechanical loading. When habitual dietary protein intake is adequate and derived from high-quality proteins providing sufficient EAA, the additional benefits of HCol supplementation on connective tissue health are likely to be limited (89).

In conclusion, current evidence suggests that HCol supplementation may provide modest benefits for selected outcomes related to skin health, joint symptoms, connective tissue, and, to a lesser extent, bone metabolism in specific populations. However, these effects are generally small, and their clinical relevance remains uncertain. The evidence is further limited by substantial heterogeneity, short intervention periods, small sample sizes, and the frequent involvement of industry-sponsored studies. Large, well-designed, independently funded, long-term randomized controlled trials controlling for baseline nutritional status, habitual protein intake, and physical activity are therefore needed to establish the clinical efficacy of HCol supplementation.

Conversely, many commercial claims (including enhanced muscle hypertrophy, athletic performance and recovery, accelerated tendon or ligament repair, osteoporosis prevention or treatment, improved gut health, hair and nail growth, and generalized anti-aging effects) remain unsupported by robust clinical evidence. From a nutritional perspective, collagen should not be considered a substitute for high-quality dietary protein. Its incomplete EAA profile, particularly the absence of tryptophan and low content of leucine and other EAAs, confers limited anabolic potential and makes collagen considerably less effective than complete proteins in stimulating postprandial muscle protein synthesis.

These findings highlight a substantial gap between the modest, context-dependent effects documented in clinical studies and the broad systemic benefits commonly promoted in commercial marketing.

### 5.4 Soy protein

Soy protein is the most extensively investigated plant-derived protein source and remains one of the few vegetable proteins classified as a high-quality complete protein because it provides all EAA required for human protein synthesis. Nevertheless, compared with animal-derived proteins, particularly whey protein, soy protein contains lower amounts of leucine and exhibits a slightly lower digestibility, characteristics that may reduce its capacity to maximally stimulate postprandial muscle protein synthesis under specific physiological conditions (90).

Current evidence indicates that the efficacy of soy protein supplementation should be interpreted according to the clinical outcome considered. While early investigations suggested broad cardiometabolic and anabolic benefits, more recent systematic re-views and meta-analyses have demonstrated that many of these effects are modest and highly dependent on the study population, supplementation protocol, and total dietary protein intake (90, 91).

The largest body of evidence regarding soy protein supplementation concerns skeletal muscle adaptations. Nowadays, WP has traditionally been considered the reference protein for maximizing postprandial muscle protein synthesis because of its higher leucine content and faster digestion kinetics (72). However, although acute studies consistently report greater stimulation of muscle protein synthesis following whey ingestion, these physiological differences are not consistently translated into superior long-term gains in muscle mass or strength. Indeed, meta-analyses suggest that soy protein supplementation can support favorable changes in body composition, although current evidence does not consistently demonstrate superiority of WP over soy protein when protein intake is adequate (36, 92). Current evidence suggests that achieving an adequate total protein intake (approximately 1.6 g·kg^−1^·day^−1^) in combination with progressive resistance exercise is a far more important determinant of muscle hypertrophy than the specific protein source (16).

The recent systematic review including 19 randomized controlled trials conducted in athletes and physically active individuals, concluded that soy protein supplementation can induce increases in lean body mass comparable to whey protein in several intervention studies, particularly when total protein intake reaches approximately 1.6 g·kg^−1^·day^−1^ or higher. Furthermore, soy protein may facilitate recovery following strenuous exercise by reducing biomarkers of muscle damage and oxidative stress, likely because of its bioactive components, including isoflavones and antioxidant phytochemicals. However, the authors also emphasized that the available evidence re-mains heterogeneous, with relatively small sample sizes and considerable variability in supplementation dose, duration, and exercise protocols (91). Definitive conclusions regarding superiority or equivalence between soy and WP cannot yet be established.

Another unresolved issue concerns endocrine responses. Although isolated studies have reported changes in circulating testosterone or cortisol concentrations following soy protein supplementation, systematic evaluation indicates that these findings are inconsistent and lack sufficient reproducibility to support clinically relevant hormonal effects. Current evidence therefore does not support the hypothesis that soy protein adversely affects the endocrine profile of healthy individuals (91).

Soy protein has been also extensively investigated for its potential cardiometabolic benefits, with the strongest and most consistent evidence relating to lipid metabolism. Metanalyses of randomized controlled trials indicate that replacing animal protein with soy protein results in modest but statistically significant reductions in total cholesterol and LDL-cholesterol, with the largest effects consistently observed in individuals with hypercholesterolemia (90, 93). The lipid-lowering effect is generally in the range of 3–5% for LDL-cholesterol, suggesting that soy protein supplementation may contribute to cardiovascular risk reduction when incorporated into an overall healthy dietary pattern, although its effect as a stand-alone nutritional intervention is relatively modest (93).

Beyond lipid metabolism, the evidence for other cardiometabolic outcomes is less consistent. Systematic reviews have reported heterogeneous findings regarding the effects of soy protein supplementation on blood pressure, insulin sensitivity, inflammatory biomarkers, triglyceride concentrations and vascular function, with considerable variability attributable to differences in study populations, intervention duration, baseline metabolic status and the quantity of soy protein and associated isoflavones administered (90, 94). Consequently, although soy protein represents an appropriate substitute for animal-derived proteins within heart-healthy dietary patterns, current evidence does not support clinically relevant benefits across all cardiometabolic endpoints (95).

In conclusion, the literature indicates that soy protein supplementation is an effective and safe strategy for increasing daily protein intake, particularly for individuals following vegetarian or vegan dietary patterns. From a nutritional perspective, soy protein should be regarded as a high-quality alternative to animal proteins rather than as a functionally superior supplement. The current body of evidence does not support claims that soy protein possesses unique anabolic or ergogenic properties beyond those expected from an adequate protein supply. Conversely, neither does it support concerns regarding detrimental hormonal effects in healthy adults (96).

Future research should prioritize large, well-controlled randomized clinical trials with standardized supplementation protocols, longer intervention periods and clinically relevant endpoints to clarify whether specific populations, including older adults, women, elite athletes and individuals with metabolic disorders, may derive greater benefits from soy protein supplementation than those observed in the general healthy population.

### 5.5 Pea and rice proteins

Among plant-derived protein supplements, pea (*Pisum sativum*) and rice (*Oryza sativa*) proteins have gained considerable attention as sustainable alternatives to ani-mal-derived proteins. Their increasing use is driven by favorable digestibility, absence of major dairy allergens, and compatibility with vegetarian and vegan dietary patterns. Nevertheless, compared with animal proteins, both pea and rice proteins exhibit limitations in EAA composition. Pea protein is relatively low in methionine and cysteine, whereas rice protein contains lower amounts of lysine. Consequently, when consumed individually, their nutritional quality is generally inferior to that of WP, although blending complementary plant proteins substantially improves AA balance and protein quality (55, 76).

The strongest evidence supporting pea and rice protein supplementation concerns skeletal muscle adaptations in physically active individuals. Randomized controlled trials comparing pea protein with WP during resistance training have consistently re-ported similar improvements in LBM, muscle thickness and maximal strength when total daily protein intake is adequate. Likewise, supplementation with rice protein isolate following resistance exercise has produced gains in body composition, muscle hypertrophy and anaerobic performance comparable to WP, particularly when relatively high doses (approximately 40–50 g per serving) are administered to compensate for the lower leucine content (97, 98). These findings suggest that total EAA intake and leucine availability, rather than protein source *per se*, are the primary determinants of long-term training adaptations.

Recent systematic reviews further support this concept while highlighting important limitations of the available evidence. Overall, plant-derived proteins appear capable of supporting resistance training-induced muscle hypertrophy and recovery, particularly when consumed in sufficient quantities or formulated as blends enriched in leucine or complementary AA. However, acute studies consistently demonstrate a lower stimulation of postprandial MPS compared with rapidly digested animal proteins, especially when isolated single-source plant proteins are provided at moderate doses. These acute physiological differences are not consistently translated into clinically meaningful differences in long-term muscle mass or strength outcomes (99, 100).

Evidence supporting the use of pea and rice protein supplementation in other physiological conditions remains considerably weaker. A limited number of intervention studies have investigated their effects on body weight regulation, appetite control and glucose homeostasis, reporting modest improvements in postprandial satiety and glycaemic responses compared with carbohydrate-based controls. Nevertheless, these findings are characterized by substantial methodological heterogeneity, relatively short intervention periods and small sample sizes, preventing definitive conclusions regarding long-term metabolic benefits (101).

Similarly, current evidence does not support specific cardioprotective effects attributable to pea or rice proteins comparable to those reported for soy protein. Improvements in circulating lipids, blood pressure, inflammatory biomarkers or insulin sensitivity have generally been inconsistent across randomized controlled trials, and no robust evidence currently indicates that supplementation with isolated pea or rice proteins confers clinically relevant cardiometabolic benefits beyond those expected from replacing less healthy dietary protein sources (55, 102).

In conclusion, from a clinical perspective, pea and rice proteins should therefore be regarded primarily as effective nutritional tools for achieving adequate daily protein intake, particularly in individuals following plant-based diets, those with dairy intolerance or allergy, or athletes seeking non-animal protein supplements. Their efficacy appears largely dependent on protein dose, AA composition and overall dietary protein intake rather than on unique bioactive properties. Future research should focus on long-term randomized controlled trials in older adults, individuals with sarcopenia, obesity and metabolic diseases, where evidence remains scarce and the potential clinical applications of these protein sources have yet to be clearly established.

## 6 Aminoacidic supplements

In this section, we focused on the scientific evidence for selected EAA-based supplements that are among the most widely consumed and commercially promoted, particularly those associated with strong health- and performance-related claims. Other AA-based supplements, including glutamine, arginine, β-alanine, creatine, and various individual AA or their derivatives, despite their widespread use in clinical and sports nutrition, were beyond the scope of the present review. This choice reflects the main objective of this narrative review: to move beyond a reductionist perspective and emphasize the importance of metabolic complexity and nutritional synergy. The biological effects of nutritional supplementation cannot be fully understood by examining individual compounds in isolation but should instead be interpreted within the broader context of nutrient interactions, complex metabolic networks, physiological status, and the overall nutritional environment.

### 6.1 Leucine and β-hydroxy-β-methyl butyrate (HMB)

Interest in supplementation with leucine and its main metabolite HMB arises from their ability to modulate muscle protein metabolism. However, it is important to distinguish the effects observed in experimental models (e.g., acute increase in MPS) from the clinically relevant benefits demonstrated in RCTs (103).

Leucine is the main EAA capable of activating MPS through the PI3K/Akt/mTORC1 pathway. Activation of mTORC1 depends on intracellular leucine recognition by Sestrin2, which, through modulation of the GATOR2 complex and Rag GTPases, promotes lysosomal recruitment and activation of mTORC1, resulting in phosphorylation of p70S6K and 4E-BP1 and initiation of protein translation (104–107). These observations constitute the molecular rationale of the “leucine trigger hypothesis,” according to which the achievement of a plasma leucine threshold represents the main determinant of the post-prandial anabolic response (108). Although leucine acutely stimulates MPS, this does not necessarily translate into long-term gains in skeletal muscle mass. Muscle hypertrophy is determined by the cumulative net protein balance, resulting from the integration of both protein synthesis, which required all AAs availability, and protein breakdown over time, whereas acute MPS measurements represent a surrogate endpoint that does not fully capture chronic adaptations (109, 110).

Systematic reviews show significant discrepancy between molecular and clinical effects. In most RCTs conducted in young, trained subjects, adding leucine to an already protein-adequate diet does not result in significant increases in muscle mass, strength, or recovery compared to a placebo. Similarly, when supplementation is combined with high-quality proteins, the additive effect is generally negligible, likely be-cause these proteins already provide optimal amounts of leucine to maximize mTORC1 activation (111). This apparent discrepancy is linked to the concept that MPS stimulation rapidly reaches a plateau and, once the threshold for activation of the anabolic signal is exceeded, further amounts of leucine do not stimulate any further increase in protein synthesis (112). Therefore, in healthy people with an adequate protein intake, supplementation with isolated leucine appears to be poorly justified.

In HCS characterized by anabolic resistance, such as aging, prolonged immobilization, and chronic diseases, the situation is partially modified. In older adults, the MPS response to AAs is attenuated, so enriching protein blends with leucine can increase the postprandial anabolic response. However, the effects on muscle mass, strength, and physical performance are modest and become clinically relevant only when supplementation is combined with resistance exercise and an adequate overall protein intake (113). In a review of 17 RCTs enrolling 1,418 subjects, leucine-isolated supplementation did not improve muscle mass and strength in elderly. However, leucine-combined supplementation including vitamin D exhibited a significant benefit for muscle strength and performance including handgrip strength and gait speed in older adults. Then, a combination of nutritional supplements would be a viable option for improving sarcopenia (114). Therefore, leucine should be considered primarily as a tool to optimize the anabolic quality of protein intake, rather than as a supplement capable of exerting independent pharmacological effects.

HMB is derived from the metabolism of leucine but has different biological properties. Only about 5% of dietary leucine is converted to HMB; therefore, supplementation allows plasma concentrations to be reached that cannot be achieved with diet alone. Unlike leucine, HMB appears to exert dual action: it promotes protein synthesis through activation of the mTORC1 pathway and attenuates proteolysis by inhibiting the ubiquitin-proteasome system and modulating autophagy. These mechanisms justify its use in hypercatabolic conditions (115–117).

The sports setting is probably the one in which HMB marketing has had the greatest influence. However, the largest available meta-analysis, which included 302 participants (18–45 years old) in body mass and composition analysis and 248 in strength analysis, concludes that HMB does not significantly improve gains in lean mass or strength achieved with resistance training in healthy young adults. The observed effects are small, often not clinically significant, and strongly influenced by the methodological quality of the studies (111). Therefore, current evidence does not support the routine use of HMB as an ergogenic supplement in athletes.

Evidence of HMB's efficacy is more favorable in older populations. A recent systematic review with meta-analysis found that HMB supplementation is associated with improved grip strength and skeletal muscle mass index (SMI) in patients with sarcopenia, while no significant benefits were found on functional performance, such as walking speed (118). Another meta-analysis found that the combination of resistance exercise and HMB supplementation resulted in modest additional benefits on physical function, particularly on Short Physical Performance Battery (SPPB) scores and, to a lesser extent, grip strength, without significant increases in appendicular muscle mass. The authors also emphasized that approximately half of the studies included presented high risk of bias, limiting the certainty of the available evidence (119). Overall, HMB therefore appears to offer an advantage primarily in hypercatabolic and/or anabolically impaired individuals rather than in metabolically healthy individuals.

The clinical setting is probably the one in which the biological rationale for HMB appears most convincing. HMB supplementation in patients undergoing surgery is associated with a reduction in the length of hospital stay and the incidence of postoperative complications, as well as improvements in some indicators of muscle mass and function. However, even in this study, the authors emphasize the high methodological heterogeneity of the studies, the frequent risk of bias, and the need for higher-quality randomized controlled trials to confirm the results (120). Therefore, in conditions characterized by cachexia, immobilization or severe systemic inflammation, HMB could contribute to limiting the loss of muscle mass rather than increasing it, consistent with its prevalent anti-catabolic effect.

In conclusion, the evidence suggests very different conclusions for leucine and HMB than those often proposed in marketing communications. For leucine, the biological benefit is unquestionable at the molecular level, but its effect appears limited when daily protein intake is already adequate. Leucine should therefore be considered a modulator of protein nutritional quality rather than a stand-alone anabolic supplement. For HMB, the evidence is strongest almost exclusively in hypercatabolic conditions and/or anabolic resistance. In healthy adults, young people, and athletes, the additional effects, compared to adequate nutrition and proper training, are modest or absent. Therefore, the pathophysiological rationale for using leucine and HMB should be related to the treatment or prevention of muscle catabolism, rather than to enhancing performance or hypertrophy in healthy subjects.

### 6.2 Branched-chain amino acids (BCAA)

BCAA, comprising leucine, isoleucine, and valine, are among the most widely consumed dietary supplements in sports nutrition. Their popularity is primarily attributable to leucine, which acts as a key regulator of the mechanistic target of rapamycin complex 1 (mTORC1), thereby acutely stimulating MPS (121). This well-established molecular mechanism has often been extrapolated to support claims of enhanced muscle hypertrophy, exercise performance, and recovery, despite the limited and inconsistent evidence from clinical trials. Although leucine is a potent anabolic signaling molecule, sustained MPS requires the availability of all EAAs, making isolated BCAA supplementation inherently limited in its capacity to support muscle anabolism (112, 122, 123).

In athletic populations, current evidence highlights a clear discrepancy between the biological plausibility of BCAA supplementation and its clinically meaningful effects. A systematic review of 24 RCTs reported negligible benefits on exercise performance and body composition, whereas the most consistent finding was a modest attenuation of delayed-onset muscle soreness (DOMS), particularly following resistance exercise (43, 124). These findings suggest that, while BCAA may slightly reduce perceived muscle soreness, their impact on functional recovery and training adaptations is minimal.

Similarly, in older adults experiencing anabolic resistance, the efficacy of BCAA supplementation appears limited and largely dependent on the formulation used. A meta-analysis of 35 RTCs reported modest improvements in muscle strength and muscle mass; however, substantial methodological heterogeneity and the high risk of bias across many included studies warrant cautious interpretation of these findings (125). More recently, a meta-analysis investigating the combined effects of resistance training and BCAA- or leucine-enriched supplementation in older adults with sarcopenia found a significant improvement only in the skeletal muscle index, with no significant effects on handgrip strength, Short Physical Performance Battery (SPPB) scores, gait speed, or total lean mass (119). Collectively, these findings indicate that the modest benefits observed are more likely attributable to resistance exercise and adequate total protein intake than to BCAA supplementation *per se*.

In conclusion, the current body of evidence does not substantiate many of the ergogenic and anabolic claims commonly associated with BCAA supplementation, including improvements in muscle mass, strength, physical performance, and recovery. In healthy individuals who achieve recommended daily protein intakes (approximately 1.2–2.0 g·kg^−1^·day^−1^ for physically active adults), isolated BCAA supplementation provides little, if any, additional clinically relevant benefit compared with high-quality protein sources or balanced EAA formulations (37, 123). Accordingly, recent evidence supports shifting nutritional strategies toward complete protein sources or EAA supplements, which not only provide the leucine-dependent anabolic stimulus but also supply the full complement of AA substrates required to sustain muscle protein synthesis and optimize adaptive responses to exercise.

### 6.3 Essential amino acids (EAA) mixtures

EAA (histidine, isoleucine, leucine, lysine, methionine, phenylalanine, threonine, tryptophan, and valine) are indispensable substrates for protein synthesis because they cannot be synthesized *de novo* in humans and must therefore be obtained through diet. In contrast, the remaining AA are classified as non-essential AA (NEAA), although several (including arginine, cysteine, glutamine, glycine, and proline) are considered conditionally essential, as endogenous synthesis may become insufficient during periods of rapid growth, pregnancy, trauma, burns, severe infections, or other catabolic conditions.

Unlike supplementation with individual AA or BCAA, balanced formulations containing all nine EAA provide the complete substrate required for *de novo* protein synthesis while simultaneously activating anabolic signaling pathways, primarily through mTORC1 (123, 126). This concept has progressively shifted scientific interest from isolated AA supplementation toward balanced EAA formulations aimed at maximizing MPS, preserving LBM, and improving metabolic resilience across both physiological and pathological conditions.

A substantial body of evidence demonstrates that free EAA mixture stimulates MPS more effectively than equivalent amounts of intact protein, largely because they produce a rapid increase in circulating EAAs concentrations and enhance AA availability at the skeletal muscle level (126–128). Importantly, maximal stimulation of MPS requires the simultaneous availability of all EAAs, as the deficiency of even a single EAA limits protein synthesis regardless of adequate leucine availability. Consequently, balanced EAA formulations appear metabolically superior to isolated BCAAs supplementation or single-AA interventions, which can activate anabolic signaling but fail to sustain protein synthesis because of inadequate substrate availability (123, 128).

The strongest clinical evidence supporting EAA supplementation derives from studies on age-related sarcopenia and anabolic resistance (22, 129–133). Multiple randomized controlled trials have shown that balanced EAA supplementation enhances MPS, preserves LBM, improves muscle strength, and promotes physical performance in older adults, particularly when combined with resistance exercise (22, 126, 127, 134). Consistent with these findings, the recent International Society of Sports Nutrition (ISSN) Position Stand concluded that EAA supplementation reliably stimulates MPS in both younger and older adults and represents an effective nutritional strategy to preserve skeletal muscle during aging and periods of reduced physical activity (134).

Beyond healthy aging, growing evidence supports the use of balanced EAA mixtures in acute and chronic hypercatabolic states. Major surgery, trauma, prolonged immobilization, intensive care unit admission, severe infections, chronic heart failure, and chronic inflammatory diseases are all characterized by accelerated proteolysis, impaired anabolic responsiveness, and progressive skeletal muscle loss. Under these conditions, adequate provision of all EAA may partially restore net protein balance by simultaneously stimulating protein synthesis and attenuating muscle protein breakdown, thereby contributing to the preservation of LBM, functional recovery, and rehabilitation outcomes (17, 135–138).

Cancer cachexia represents another clinical setting in which balanced EAA supplementation has attracted increasing attention. Cachexia is characterized by systemic inflammation, profound anabolic resistance, increased resting energy expenditure, and progressive skeletal muscle wasting that cannot be fully reversed by conventional nutritional support alone (139–141). Although no nutritional intervention is sufficient to counteract cachexia as a standalone therapy, accumulating evidence suggests that balanced EAAs supplementation may improve nitrogen balance, attenuate muscle protein loss, and support physical function when incorporated into multimodal interventions that include exercise, nutritional counseling, and anti-inflammatory therapies (30, 142–145). Nevertheless, the available evidence remains heterogeneous, and adequately powered RCTs are required before routine clinical implementation can be recommended (146).

An emerging area of interest is in subjects with prolonged bed rest and/or immobilization. Experimental studies consistently demonstrate that even short periods of physical inactivity suppress MPS and accelerate skeletal muscle atrophy. In this context, EAA supplementation appears to partially preserve anabolic responsiveness and attenuate lean tissue loss, particularly when combined with early mobilization and rehabilitation strategies. These findings may have important clinical implications for hospitalized older adults, postoperative patients, and critically ill individuals, in whom muscle wasting is strongly associated with prolonged disability and adverse clinical outcomes (127, 133).

Beyond their anabolic effects mediated through mTORC1 activation, balanced EAA formulations may exert broader metabolic actions. Experimental evidence suggests that EAA promote mitochondrial biogenesis, improve mitochondrial protein turnover, support antioxidant defenses, enhance insulin signaling, and preserve metabolic flexibility. Additional studies have also reported favorable effects on immune function, wound healing, and recovery from catabolic stress, although these mechanisms require further clinical confirmation (121, 147–149).

Despite these promising findings, several limitations should be acknowledged. Considerable heterogeneity exists among published studies with respect to EAA composition, dosage (generally ranging from 8 to 20 g/day), duration of supplementation, study populations, and concomitant nutritional or exercise interventions. Moreover, many RCTs are limited by relatively small sample sizes and focus primarily on surrogate outcomes, such as MPS or LBM, rather than clinically meaningful endpoints including functional independence, hospitalization, quality of life, or mortality. Therefore, although current evidence strongly supports the anabolic efficacy of balanced EAA supplementation, further large-scale RCTs are needed to establish optimal formulations, dosing regimens, treatment duration, and the patient populations most likely to benefit. Table 7 summarized the differences between main commercial claim and scientific evidence regarding the most common protein/AA supplements.

**Table 7:** A summary of the main characteristics, commercial claims, scientific evidence, and actual indications regarding the main protein and amino acid supplements.

Product	Main features	Commercial claim	Scientific evidence	Real indications	Notes
Whey protein	High EAAs content (~50%); leucine ~10–12%; fast digestion; DIAAS >1.0	Rapidly increase muscle mass and strength	High	Post-exercise recovery; sarcopenia prevention; clinical nutritional support	Real benefit, but not superior to a proper diet. Differences between concentrates, isolates, and hydrolyzed products are often overestimated.
Casein	Slow digestion; prolonged release of AA; good biological quality	Prolonged muscle protection	Moderate	support night fasting; maintain lean mass	Below WP for acute MPS stimulus
Collagen	Incomplete EAAs profile, absence of tryptophan, low content of leucine and other EAAs	Skin health, muscle hypertrophy, performance, tendon repair, bone health, gut health, hair growth, and anti-aging	Low and very low	Very low improvements in skin hydration and elasticity after long treatment combined with mechanical loading or resistance exercise	High evidence against claim
Soy	Complete AA profile but lower leucine than WP	Equivalent to animal proteins	Moderate	Vegan diets; food intolerances	Lower anabolic power
Pea/Rice	Complementary AA profiles	Equivalent to animal proteins	Low to moderate	Plant-based protein alternative	Fortification with EAA or leucine required
EAA-mix	All 9 EAAs in stoichiometric ratio	They activate protein synthesis even at low doses	High	Elderly; sarcopenia; hypercatabolic patients; ICU patients; low-calorie diets; intensive training.	Often mistakenly assimilated and commercially confused with BCAA
BCAAs	Leucine, isoleucine, valine	They stimulate MPS	Low	No primary indication	Do not stimulate complete MPS due to a lack of other EAA
Leucine	Activates mTORC1 and MPS	Anabolism switch	Moderate	Protein/EAA support	Ineffective on its own
HMB	Anti-catabolic leucine metabolite	Prevents muscle loss	Moderate (context-specific)	Geriatric and/or immobilized patients	Non-univocal evidence

MPS, muscle protein synthesis; DIAAS, Digestible Indispensable-AA-Score.

## 7 Potential risks associated with the indiscriminate use of protein and amino acid supplements

The use of protein and AAs supplements has increased substantially over the last decade, extending well beyond elite athletes to recreational exercisers, older adults, adolescents, and individuals seeking weight management or aesthetic benefits. While protein supplementation can be beneficial when dietary protein intake is insufficient or physiological requirements are increased, indiscriminate or excessive supplementation is not supported by current evidence and may expose susceptible individuals to unnecessary health risks without providing additional physiological benefits (37, 46, 150).

Skeletal MPS reaches a plateau once an adequate amount of high-quality protein containing sufficient leucine has been consumed. Additional protein intake above this anabolic threshold does not proportionally increase MPS but is instead largely oxidized, contributing to ureagenesis and nitrogen excretion (16, 46). Consequently, chronic protein supplementation in individuals already meeting their daily protein requirements provides minimal additional benefit for muscle hypertrophy or strength adaptations.

Recent evidence further indicates that no tolerable upper intake level has yet been established for dietary protein because long-term prospective studies evaluating chronic excessive protein consumption remain scarce. Consequently, although high protein intakes appear safe in healthy individuals over the short to medium term, uncertainty persists regarding lifelong exposure to excessive supplementation (150).

Renal safety remains one of the most debated issues surrounding protein supplementation. Current evidence indicates that high-protein diets do not appear to impair kidney function in healthy adults. Increased glomerular filtration rate and renal blood flow observed during high protein intake are generally considered physiological adaptive responses rather than indicators of renal injury (151, 152). The situation differs markedly in individuals with pre-existing chronic kidney disease (CKD). The updated KDIGO 2024 Clinical Practice Guideline recommends a protein intake of approximately 0.8 g/kg/day in adults with CKD stages G3–G5 and advises avoiding protein intakes exceeding 1.3 g/kg/day in those at risk of disease progression. Importantly, low- or very-low-protein diets should not be prescribed in metabolically unstable patients, particularly when malnutrition, sarcopenia, or cachexia is present (153–155). Therefore, indiscriminate protein supplementation should be discouraged in individuals with impaired renal function unless specifically prescribed under medical supervision.

The pioneering study by Giovannetti and Maggiore in patients with CKD provides an excellent historical example of how an apparently reductionist strategy (reducing dietary protein intake) was in fact grounded in an integrated understanding of metabolism. The underlying message was not simply “eat less protein”, but rather “provide the minimum amount of high-quality protein required to maintain protein homeostasis while ensuring adequate energy intake.” Their work demonstrated that not all proteins are metabolically equivalent and that protein requirements depend not only on the quantity consumed but also on protein quality and the individual's metabolic context (156). These concepts anticipated several principles that are now central to modern protein nutrition, including the importance of protein quality, EAA availability, the protein-sparing effect of adequate energy intake, and the recognition that the efficacy of nutritional interventions emerges from the interaction among protein composition, energy supply, and the organism's metabolic state, rather than from the amount of protein considered in isolation.

Isolated AA supplementation also deserves careful consideration. BCAA, particularly leucine, has long been marketed as anabolic supplements. However, recent evidence confirms that although BCAA activates mTOR signaling and transiently stimulates muscle protein synthesis, their anabolic effect is substantially inferior to that achieved by complete protein sources providing the full complement of EAA (123). An updated review published in Nutrition Research Reviews concluded that BCAA supplementation alone cannot sustain maximal muscle protein synthesis because all EAA are required for continued protein accretion. Consequently, supplementation with isolated BCAA should not replace the consumption of complete dietary proteins or EAA-mixtures.

Growing attention has also been directed toward the metabolic implications of chronic AA supplementation. Elevated circulating BCAA concentrations have consistently been associated with insulin resistance, obesity, non-alcoholic fatty liver disease, and type 2 diabetes. Nevertheless, current evidence suggests that these associations primarily reflect altered AA metabolism secondary to metabolic dysfunction rather than direct toxicity of BCAA supplementation itself (157, 158). Recent reviews continue to emphasize that causality has not been established and that further long-term randomized clinical trials are required before definitive conclusions can be drawn.

Other AA supplements, including glutamine, arginine, β-alanine and creatine, have demonstrated favorable safety profiles when consumed within recommended dosages. Nevertheless, recent comprehensive reviews emphasize that excessive chronic intake, unsupervised combinations of multiple supplements, or supplementation in individuals with hepatic or renal disorders may increase the risk of adverse metabolic consequences, highlighting the need for individualized nutritional assessment before supplementation.

Increasingly recognized concern involves supplement quality rather than protein intake *per se*. Unlike pharmaceutical products, dietary supplements are subject to relatively limited regulatory oversight in many countries. Consequently, variability in protein content, AA composition, microbiological quality, and labeling accuracy have been repeatedly documented. Furthermore, contamination with heavy metals, anabolic steroids, stimulants, or substances prohibited by the World Anti-Doping Agency (WADA) continues to represent an important issue, particularly for competitive athletes (159, 160). Recent analytical studies have detected measurable concentrations of lead, cadmium, and other elemental contaminants in some commercially available protein supplements, underscoring the importance of rigorous manufacturing practices, independent third-party testing, and quality certification to ensure product safety and consumer confidence (161, 162).

Excessive reliance on protein supplements may inadvertently reduce the consumption of whole-food protein sources. Whole foods provide not only EAA but also vitamins, minerals, dietary fiber, essential fatty acids, and numerous bioactive compounds that collectively contribute to maintaining the metabolic complexity and so body health. Accordingly, current sports nutrition guidelines consistently emphasize that dietary supplements should complement rather than replace a balanced diet and that supplementation should be individualized according to age, health status, physical activity level, and specific nutritional requirements (37, 163).

Finally, recent review highlights that AA supplementation should be considered within the broader context of dietary protein intake, physiological requirements, and specific metabolic or pathophysiological conditions, rather than as an isolated nutritional intervention. Potential benefits must be balanced against safety considerations, with supplementation guided by evidence-based requirements and upper safe intake levels (164).

In conclusion, current evidence indicates that protein and AA supplements are generally safe when consumed appropriately by healthy individuals within evidence-based recommendations. However, indiscriminate supplementation, prolonged excessive intake, reliance on isolated AA instead of complete protein sources, and the use of poorly regulated products may expose susceptible individuals to avoidable health risks while providing little or no additional physiological benefit beyond that achievable through an adequate and balanced dietary intake (150).

## 8 Conclusions

Considering the available preclinical and clinical evidence, protein and AA supplementation should not be regarded as universally necessary or routinely recommended. Rather, **there is stronger evidence for protein supplementation when total dietary intake is insufficient or anabolic requirements are increased**. The discrepancy between commercial claims and scientific evidence largely arises from the inappropriate extrapolation of findings obtained in selected populations, such as older adults with anabolic resistance, patients with catabolic conditions, or highly trained athletes, to the general healthy population, in whom the expected benefits are often limited or absent.

When supplementation is indicated based on patients' personalized needs, **there is moderate evidence supporting WP, EAA**, **β-HMB in selected populations**, as high-quality complete protein sources or balanced formulations containing all EAA provide both the anabolic stimulus and the full complement of substrates required to sustain muscle protein synthesis. In contrast, **there is weaker evidence for isolated BCAA in healthy, protein-sufficient individuals**, as supplementation with individual AA or incomplete AA blends has generally failed to demonstrate clinically meaningful advantages over complete protein or EAA formulations. Furthermore, **there is currently limited and outcome-specific evidence for collagen supplementation**. Consequently, their widespread use appears to be driven more by commercial positioning than by robust physiological or clinical evidence. In summary, as outlined and summarized in Figure 1, integrating reductionist knowledge with systems complexity provides a more realistic framework for assessing the efficacy of dietary supplements, understanding biological variability, and improving the translational impact of nutritional research and clinical practice.

## References

[1] Kwon O. A big picture view of precision nutrition: from reductionism to holism. J Nutr Health. (2019) 52:1–5. doi: 10.4163/jnh.2019.52.1.1

[2] Mazzocchi F. Complexity in biology. EMBO Rep. (2008) 9:6. doi: 10.1038/sj.embor.7401147 PMC2246621 18174892

[3] Cordain L Eaton SB Sebastian A Mann N Lindeberg S Watkins BA O'Keefe JH Brand-Miller J. Origins and evolution of the Western diet: health implications for the 21st century. Am J Clin Nutr. (2005) 81:341–54. doi: 10.1093/ajcn.81.2.341 15699220

[4] Fardet A Rock E. Toward a new philosophy of preventive nutrition: from a reductionist to a holistic paradigm to improve nutritional recommendations. Adv Nutr. (2014) 5, 430–46. doi: 10.3945/an.114.006122 PMC4085191 25022992

[5] Kitano H. Systems biology: a brief overview. Science. (2002) 295:1662–4. doi: 10.1126/science.1069492 11872829

[6] Barabási AL Oltvai ZN. Network biology: understanding the cell's functional organization. Nat Rev Genet. (2004) 5:101–13. doi: 10.1038/nrg1272 14735121

[7] Simpson SJ Le Couteur DG Small L Brandon AE Raubenheimer D. Macronutrient mixtures and interactions in health and disease. Nat Rev Endocrinol. (2026). doi: 10.1038/s41574-026-01266-5 42331991

[8] Townsend JR Kirby TO Sapp PA Gonzalez AM Marshall TM Esposito R. Nutrient synergy: definition, evidence, and future directions. Front Nutr. (2023) 10:1279925. doi: 10.3389/fnut.2023.1279925 PMC10600480 37899823

[9] Morin E. “On complexity,” in Advances in Systems Theory, Complexity, and the Human Sciences (Cresskill, NJ: Hampton Press) (2008)

[10] Williamson EM. Synergy and other interactions in phytomedicines. Phytomedicine. (2001) 8:401–9. doi: 10.1078/0944-7113-00060 11695885

[11] Ioannidis JPA. The challenge of reforming nutritional epidemiologic research. JAMA. (2018) 320:969–70. doi: 10.1001/jama.2018.11025 30422271

[12] Heylighen F Cilliers P Gershenson C. “Complexity and philosophy,” in Complexity, Science and Society, eds. Bogg J Geyer R [arXiv:cs/0604072v1] (Oxford: Radcliffe Publishing) (2007).

[13] O'Brien BC Harris IB Beckman TJ Reed DA Cook DA. Standards for reporting qualitative research: a synthesis of recommendations. Acad Med. (2014) 89:1245–51. doi: 10.1097/ACM.0000000000000388 24979285

[14] EFSA EFSA Panel on Dietetic Products Nutrition and Allergies (NDA). Dietary reference values for nutrients: summary report. EFSA Support Publ. (2017) 14:e15121. doi: 10.2903/j.efsa.2016.4484

[15] Garthe I Maughan RJ. Athletes and supplements: a practical approach. Int J Sport Nutr Exerc Metab. (2020) 30:249–51. 10.1123/ijsnem.2017-0429 29580114

[16] Morton RW Murphy KT McKellar SR Schoenfeld BJ Henselmans M Helms E . A systematic review, meta-analysis and meta-regression of the effect of protein supplementation on resistance training-induced gains in muscle mass and strength in healthy adults. Br J Sports Med. (2018) 52:376–84. doi: 10.1136/bjsports-2017-097608 PMC5867436 28698222

[17] Corsetti G Pasini E. Amino acids as metabokines in hypercatabolic states: rethinking nutritional protein-based strategies be-yond caloric support. Nutrients. (2026) 18:1703. doi: 10.3390/nu18111703 PMC13257804 42280346

[18] Deutz NEP Bauer JM Barazzoni R Biolo G Boirie Y Bosy-Westphal A . Protein intake and exercise for optimal muscle function with aging: recommendations from the ESPEN Expert Group. Clin Nutr. (2014) 33:929–936. doi: 10.1016/j.clnu.2014.04.007 PMC4208946 24814383

[19] Bauer J Biolo G Cederholm T Cesari M Cruz-Jentoft AJ Morley JE . Evidence-based recommendations for optimal dietary protein intake in older people: A position paper from the PROT-AGE Study Group. J Am Med Dir Assoc (2013) 14:542–59. doi: 10.1016/j.jamda.2013.05.021 23867520

[20] Rondanelli M Klersy C Terracol G Talluri J Maugeri R Guido D . Whey protein, amino acids, and vitamin D supplementation with physical activity increases fat-free mass and strength, functionality, and quality of life and decreases inflammation in sarcopenic elderly. Am J Clin Nutr. (2016) 103:830–40. doi: 10.3945/ajcn.115.113357 26864356

[21] Rondanelli M Peroni G Gasparri C Infantino V Nichetti M Cuzzoni G . Is a combination of melatonin and amino acids useful to sarcopenic elderly patients? A randomized trial. Geriatrics. (2019) 4:4. doi: 10.3390/geriatrics4010004 PMC6473373 31023972

[22] Rondanelli M Opizzi A Antoniello N Boschi F Iadarola P Pasini E . Effect of essential amino acid supplementation on quality of life, amino acid profile and strength in institutionalized elderly patients. Clin Nutr. (2011) 30:571–7. doi: 10.1016/j.clnu.2011.04.005 21636183

[23] Cheng H Kong J Underwood C Petocz P Hirani V Dawson B . Systematic review and meta-analysis of the effect of protein and amino acid supplements in older adults with acute or chronic conditions. Br J Nutr. (2018) 119:527–42. doi: 10.1017/S0007114517003816 29508691

[24] Ten Haaf DSM Eijsvogels TMH Bongers CCWG Horstman AMH Timmers S de Groot LCPGM . Protein supplementation improves lean body mass in physically active older adults: a randomized placebo-controlled trial. J Cachexia Sarcopenia Muscle. (2019) 10:298–310. doi: 10.1002/jcsm.12394 PMC6463466 30848096

[25] Xu ZR Tan ZJ Zhang Q Gui QF Yang YM. Clinical effectiveness of protein and amino acid supplementation on building muscle mass in elderly people: a meta-analysis. PLoS ONE. (2014) 9:e109141. doi: 10.1371/journal.pone.0109141 PMC4182521 25268791

[26] Xie C Yan R Tao R. Combined resistance training and amino acid-based supplementation for sarcopenia in older adults: a systematic review and meta-analysis. BMC Musculoskelet Disord. (2026) 27:35. doi: 10.1186/s12891-025-09436-8 PMC12809905 41540398

[27] Yoshimura Y Matsumoto A Inoue T Okamura M Kuzuya M. Protein supplementation alone or combined with exercise for sarcopenia and physical frailty: a systematic review and meta-analysis of randomized controlled trials. Arc Geronto Geriat. (2025) 131:105783. doi: 10.1016/j.archger.2025.105783 39955964

[28] Parrott J Frank L Rabena R Craggs-Dino L Isom KA Greiman L. American society for metabolic and bariatric surgery integrated health nutritional guidelines for the surgical weight loss patient-−2016 update: micronutrients. Surg Obes Relat Dis. (2017) 13:727–41. doi: 10.1016/j.soard.2016.12.018 28392254

[29] Bonnefoy M Laville M Ecochard R Jusot JF Normand S Maillot S . Effects of branched amino acids supplementation in malnourished elderly with catabolic status. J Nutr Health Aging. (2010) 14:579–84. doi: 10.1007/s12603-010-0090-1 PMC12880318 20818474

[30] Ragni M Fornelli C Nisoli E Penna F. Amino acids in cancer and cachexia: an integrated view. Cancers. (2022) 14:5691. doi: 10.3390/cancers14225691 PMC9688864 36428783

[31] Veldsman L Richards GA Nel D Kohn TA Blaauw R. Combined intravenous bolus amino acid supplementation and mobilization on early muscle loss in critically ill adults: a randomized controlled trial. JPEN J Parenter Enteral Nutr. (2026) 50:170–82. doi: 10.1002/jpen.70041 PMC12865756 41472548

[32] Heming N Carlier R Prigent H Mekki A Jousset C Lofaso F . Effect of an enteral amino acid blend on muscle and gut functionality in critically ill patients: a proof-of-concept randomized controlled trial. Crit Care (2022) 26:358. doi: 10.1186/s13054-022-04232-5 PMC9670468 36397118

[33] Wandrag L Brett SJ Frost G Hickson M. Impact of supplementation with amino acids or their metabolites on muscle wasting in patients with critical illness or other muscle wasting illness: a systematic review. J Hum Nutr Diet. (2015) 28:313–30. doi: 10.1111/jhn.12238 24807079

[34] Wittholz K Bidgood E Fetterplace K McLean A Rooyackers O Deane AM . A systematic review to assess the impact of amino acids or their derivatives on skeletal muscle wasting in critically ill patients. Clin Nutr. (2024) 43:2458–72. doi: 10.1016/j.clnu.2024.09.025 39305756

[35] Bels JLM Abdelhamid YA van de Poll MCG. Protein supplementation in critical illness: Why, when and how? Curr Opin Clin Nutr Metab Care. (2023) 26:146–53. doi: 10.1097/MCO.0000000000000912 36728596

[36] Morgan PT Harris DO Marshall RN Quinlan JI Edwards SJ Allen SL . Protein source and quality for skeletal muscle anabolism in young and older adults: a systematic review and meta-analysis. J Nutr. (2021) 151:1901–20. doi: 10.1093/jn/nxab055 PMC8245874 33851213

[37] Jäger R Kerksick CM Campbell BI Cribb PJ Wells SD Skwiat TM . International society of sports nutrition position stand: protein and exercise. J Int Soc Sports Nutr. (2017) 14:20. doi: 10.1186/s12970-017-0177-8 PMC5477153 28642676

[38] Moughan PJ Lim WXJ. Digestible Indispensable Amino Acid Score (DIAAS): 10 years on. Front Nutr. (2024) 11:1389719. doi: 10.3389/fnut.2024.1389719 PMC11252030 39021594

[39] Kerksick CM Wilborn CD Roberts MD Smith-Ryan A Kleiner SM Jäger R . ISSN exercise and sports nutrition review update: re-search and recommendations. J Int Soc Sports Nutr. (2018) 15:38. doi: 10.1186/s12970-018-0242-y PMC6090881 30068354

[40] Master PBZ Macedo RCO. Effects of dietary supplementation in sport and exercise: a review of evidence on milk proteins and amino acids. Crit Rev Food Sci Nutr. (2021) 61:1225–39. doi: 10.1080/10408398.2020.1756216 32363897

[41] Lam FC Khan TM Faidah H Haseeb A Khan AH. Effectiveness of whey protein supplements on the serum levels of amino acid, creatinine kinase and myoglobin of athletes: a systematic review and meta-analysis. Syst Rev. (2019) 8:130. doi: 10.1186/s13643-019-1039-z PMC6544960 31151484

[42] Park S Church DD Azhar G Schutzler SE Ferrando AA Wolfe RR. Anabolic response to essential amino acid plus whey protein composition is greater than whey protein alone in young healthy adults. J Int Soc Sports Nutr. (2020) 17:9. doi: 10.1186/s12970-020-0340-5 PMC7011510 32041644

[43] Martinho DV Nobari H Faria A Field A Duarte D Sarmento H. Oral branched-chain amino acids supplementation in athletes: a systematic review. Nutrients. (2022) 14:4002. doi: 10.3390/nu14194002 PMC9571679 36235655

[44] Marcon M Zanella PB. The effect of branched-chain amino acids supplementation in physical exercise: a systematic review of human randomized controlled trials. Sci Sports. (2022) 37:393–404. doi: 10.1016/j.scispo.2021.05.006

[45] Plotkin DL Delcastillo K Van Every DW Tipton KD Aragon AA. Isolated leucine and branched-chain amino acid supplementation for enhancing muscle protein synthesis and muscle mass: a review. Int J Sport Nutr Exerc Metabol (2021) 31, 292–301. doi: 10.1123/ijsnem.2020-0356 33741748

[46] Phillips SM van Loon LJC. Dietary protein for athletes: from requirements to optimum adaptation. J Sports Sci (2011) 29:S29–38. doi: 10.1080/02640414.2011.619204 22150425

[47] Berrazaga I Micard V Gueugneau M Walrand S. The role of the anabolic properties of plant- vs. animal-based protein sources in supporting muscle mass maintenance: a critical review. Nutrients. (2019) 11:1825. doi: 10.3390/nu11081825 PMC6723444 31394788

[48] Food and Agriculture Organization of the United Nations (FAO). “Dietary protein quality evaluation in human nutrition: report of an fao expert consultation,” Fao Food and Nutrition Paper No. 92 (Rome: FAO) (2013), 1–66. 26369006

[49] Maughan RJ Burke LM Dvorak J Larson-Meyer DE Peeling P Phillips SM . IOC consensus statement: dietary supplements and the high-performance athlete. Br J Sports Med (2018) 52:439–55. doi: 10.1136/bjsports-2018-099027 PMC5867441 29540367

[50] Wolfe RR Church DD Ferrando AA Moughan PJ. Consideration of the role of protein quality in determining dietary protein recommendations. Front Nutr. (2024) 11:1389664. doi: 10.3389/fnut.2024.1389664 PMC11598328 39606577

[51] Karoui R Bouaicha I. A review on nutritional quality of animal and plant-based milk alternatives: a focus on protein. Front Nutr. (2024) 11:1378556. doi: 10.3389/fnut.2024.1378556 PMC11259050 39036491

[52] Sá AGA House JD. “Nutritional properties: protein quality assessment parameters and methods,” in Functionality of Plant Proteins, eds. Wanasundara JPD Schmitt C Lamsal BP (Cambridge, MA, USA: Academic Press) (2024), 253–73. doi: 10.1016/B978-0-323-91721-6.00011-6

[53] Melina V Craig W Levin S. Position of the academy of nutrition and dietetics: vegetarian diets. J Acad Nutr Diet. (2016) 116:1970–80. doi: 10.1016/j.jand.2016.09.025 27886704

[54] Medina-Vera I Avila-Nava A León-López L Gutiérrez-Solis AL Talamantes-Gómez JM Márquez-Mota CC. Plant-based proteins: clinical and technological importance. Food Sci Biotechnol. (2024) 33:2461–75. doi: 10.1007/s10068-024-01600-5 PMC11319542 39144188

[55] van Vliet S Burd NA van Loon LJC. The skeletal muscle anabolic response to plant- vs. animal-based protein con-sumption. J Nutr. (2015) 145:1981–91. doi: 10.3945/jn.114.204305 26224750

[56] Castro ALH de Araújo FHS Olimpio MYM B de B Primo R Pereira TT Lopes LAF . Comparative meta-analysis of the effect of concentrated, hydrolyzed, and isolated whey protein supplementation on body composition of physical activity practitioners. Nutrients. (2019) 11:2047. doi: 10.3390/nu11092047 PMC6769754 31480653

[57] Tang C Xi T Zheng J Cui X. Chemical properties of whey protein in protein powders and its impact on muscle growth in athletes: a review. Nat Prod Commun. (2025) 20:1–8. doi: 10.1177/1934578X251326124

[58] Sepandi M Samadi M Shirvani H Alimohamadi Y Taghdir M Goudarzi F . Effect of whey protein supplementation on weight and body composition indicators: a meta-analysis of randomized clinical trials. Clin Nutr ESPEN (2022) 50:74–83. doi: 10.1016/j.clnesp.2022.05.020 35871954

[59] Al-Rawhani AH Adznam SNA Abu Zaid Z Md Yusop NB Sallehuddin HM Alshawsh MA. Effectiveness of whey protein supplementation on muscle strength and physical performance of older adults: a systematic review and meta-analysis of randomized clinical trials. Clin Nutr. (2024) 43:2412–26. doi: 10.1016/j.clnu.2024.08.033 39303495

[60] Zhang L Liu G Huang X He F. Effects of protein supplementation on muscle mass, muscle strength, and physical perfor-mance in older adults with physical inactivity: a systematic review and meta-analysis. BMC Geriatr. (2025) 25:228. doi: 10.1186/s12877-025-05885-x PMC11978179 40200135

[61] Amirani E Milajerdi A Reiner Ž Mirzaei H Mansournia MA Asemi Z. Effects of whey protein on glycemic control and serum lipoproteins in patients with metabolic syndrome and related conditions: a systematic review and meta-analysis of randomized controlled clinical trials. Lipids Health Dis. (2020) 19:209. doi: 10.1186/s12944-020-01384-7 PMC7504833 32958070

[62] Prokopidis K Morgan PT Veronese N Morwani-Mangnani J Triantafyllidis KK Kechagias KS . The effects of whey protein supplementation on indices of cardiometabolic health: a systematic review and meta-analysis of randomized controlled trials. Clin Nutr (2025) 44:109–21. doi: 10.1016/j.clnu.2024.12.003 39647241

[63] Zhang JW Tong X Wan Z Wang Y Qin LQ Szeto IM. Effect of whey protein on blood lipid profiles: a meta-analysis of randomized controlled trials. Eur J Clin Nutr (2016) 70:879–85. doi: 10.1038/ejcn.2016.39 27026427

[64] Zhou LM Xu JY Rao CP Han S Wan Z Qin LQ. Effect of whey supplementation on circulating C-reactive protein: a meta-analysis of randomized controlled trials. Nutrients. (2015) 7:1131–43. doi: 10.3390/nu7021131 PMC4344580 25671415

[65] Orsso CE Caretero A Poltronieri TS Arends J van der Schueren MAE Kiss N . Effects of high-protein supplementation during cancer therapy: a systematic review and meta-analysis. Am J Clin Nutr (2024) 120, 1311–24. doi: 10.1016/j.ajcnut.2024.08.016 PMC11619795 39631998

[66] Rabie ASI Alhomsi T AbouKhatwa MM Mosilhy EA Elzahaf RA. Impact of whey protein supplementation as adjuvant therapy on mal-nourished cancer patients: systematic review and meta-analysis. Discov Food (2024) 4:118. doi: 10.1007/s44187-024-00171-y

[67] Vasconcelos QDJS Bachur TPR Aragão GF. Whey protein supplementation and its potentially adverse effects on health: a systematic review. Appl Physiol Nutr Metab. (2021) 46:27–33. doi: 10.1139/apnm-2020-0370 32702243

[68] Boirie Y Dangin M Gachon P Vasson MP Maubois JL Beaufrère B. Slow and fast dietary proteins differently modulate postprandial protein accretion. Proc Natl Acad Sci USA. (1997) 94:14930–5. doi: 10.1073/pnas.94.26.14930 PMC25140 9405716

[69] Res PT Groen B Pennings B Beelen M Wallis GA Gijsen AP . Protein ingestion before sleep improves postexercise overnight recovery. Med Sci Sports Exerc. (2012) 44:1560–9. doi: 10.1249/MSS.0b013e31824cc363 22330017

[70] Trommelen J van Loon LJC. Pre-sleep protein ingestion to improve the skeletal muscle adaptive response to exercise training. Nutrients. (2016) 8:763. doi: 10.3390/nu8120763 PMC5188418 27916799

[71] Dela Cruz J Kahan D. Pre-sleep casein supplementation, metabolism, and appetite: a systematic review. Nutrients. (2021) 13:1872. doi: 10.3390/nu13061872 PMC8229753 34070862

[72] Tang JE Moore DR Kujbida GW Tarnopolsky MA Phillips SM. Ingestion of whey hydrolysate, casein, or soy protein isolate: effects on mixed muscle protein synthesis at rest and following resistance exercise in young men. J Appl Physiol. (2009) 107:987–92. doi: 10.1152/japplphysiol.00076.2009 19589961

[73] Pennings B Boirie Y Senden JM Gijsen AP Kuipers H van Loon LJC. Whey protein stimulates postprandial muscle protein accretion more effectively than do casein and casein hydrolysate in older men. Am J Clin Nutr. (2011) 93:997–1005. doi: 10.3945/ajcn.110.008102 21367943

[74] Reidy PT Rasmussen BB. Role of ingested amino acids and protein in the promotion of resistance exercise-induced muscle protein anabolism. J Nut. (2016) 146:155–83. doi: 10.3945/jn.114.203208 PMC4725426 26764320

[75] Zhou HH Liao Y Zhou X Peng Z Xu S Shi S . Effects of timing and types of protein supplementation on improving muscle mass, strength, and physical performance in adults undergoing resistance training: a network meta-analysis. Int J Sport Nutr Exerc Metab (2024) 34:54–64. doi: 10.1123/ijsnem.2023-0118 38039960

[76] Gorissen SHM Crombag JJR Senden JMG Waterval WAH Bierau J Verdijk LB . Protein content and amino acid composition of commercially available plant-based protein isolates. Amino Acids (2018) 50:1685–95. doi: 10.1007/s00726-018-2640-5 PMC6245118 30167963

[77] Breen L Phillips SM. Skeletal muscle protein metabolism in the elderly: interventions to counteract the anabolic resistance of ageing. Nutr Metab. (2011) 8:68. doi: 10.1186/1743-7075-8-68 PMC3201893 21975196

[78] Aragon AA Tipton KD Schoenfeld BJ. Age-related muscle anabolic resistance: inevitable or preventable? Nutr Rev. (2023) 81:441–54. doi: 10.1093/nutrit/nuac062 36018750

[79] Romano C Corsetti G Flati V Pasini E Picca A Calvani R . Influence of diets with varying essential/nonessential amino acid ratios on mouse lifespan. Nutrients (2019) 11:1367. doi: 10.3390/nu11061367 PMC6628056 31216646

[80] Cermak NM Res PT de Groot LCPGM Saris WHM van Loon LJC. Protein supplementation augments the adaptive response of skeletal muscle to resistance-type exercise training: a meta-analysis. Am J Clin Nutr. (2012) 96:1454–64. doi: 10.3945/ajcn.112.037556 23134885

[81] Messina M Lynch H Dickinson JM Reed KE. No difference between the effects of supplementing with soy protein vs. animal protein on gains in muscle mass and strength in response to resistance exercise. Int J Sport Nutr Exerc Metab. (2018) 28:674–85. doi: 10.1123/ijsnem.2018-0071 29722584

[82] Reis CEG Loureiro LMR Roschel H da Costa THM. Effects of pre-sleep protein consumption on muscle-related out-comes: a systematic review. J Sci Med Sport. (2021) 24:177–82. doi: 10.1016/j.jsams.2020.07.016 32811763

[83] Pu SY Huang YL Pu CM Kang YN Hoang KD Chen KH . Effects of oral collagen for skin anti-aging: a systematic review and meta-analysis. Nutrients. (2023) 15:2080. doi: 10.3390/nu15092080 PMC10180699 37432180

[84] Campos LD Santos Junior VA Pimentel JD Carregã GLF Cazarin CBB. Collagen supplementation in skin and orthopedic diseases: a review of the literature. Heliyon. (2023) 9:e14961. doi: 10.1016/j.heliyon.2023.e14961 PMC10102402 37064452

[85] Ravindran R Pizzol D López-Gil JF Rahmati M Boyer L Fond G . Collagen supplementation for skin and musculoskeletal health: an umbrella review of meta-analyses on elasticity, hydration, and structural outcomes. Aesthet Surg J Open Forum. (2026) 8:ojag018. doi: 10.1093/asjof/ojag018 PMC12968778 41809116

[86] Khatri M Naughton RJ Clifford T Harper LD Corr L. The effects of collagen peptide supplementation on body composition, collagen synthesis, and recovery from joint injury and exercise: a systematic review. Amino Acids. (2021) 53:1493–506. doi: 10.1007/s00726-021-03072-x PMC8521576 34491424

[87] Myung SK Park Y. Effects of collagen supplements on skin aging: a systematic review and meta-analysis of randomized controlled trials. Am J Med. (2025) 138:1264–77. doi: 10.1016/j.amjmed.2025.04.034 40324552

[88] Bassila C Bassila J-C Slim M. Efficacy and safety of hydrolyzed collagen supplementation on skin health outcomes: a systematic literature review of randomized controlled trials. Eur J Clin Nutr. (2026). doi: 10.1038/s41430-026-01778-3 42342959

[89] Holwerda AM van Loon LJC. The impact of collagen protein ingestion on musculoskeletal connective tissue remodeling: a narrative review. Nutr Rev. (2022) 80:1497–514. doi: 10.1093/nutrit/nuab083 PMC9086765 34605901

[90] Messina M. Soy and health update: evaluation of the clinical and epidemiologic literature. Nutrients. (2016) 8:754. doi: 10.3390/nu8120754 PMC5188409 27886135

[91] Zare R Devrim-Lanpir A Guazzotti S Ali Redha A Prokopidis K Spadaccini D . Effect of soy protein supplementation on muscle adaptations, metabolic and antioxidant status, hormonal response, and exercise performance of active individuals and athletes: a systematic review of randomized controlled trials. Sports Med. (2023) 53:2417–46. doi: 10.1007/s40279-023-01899-w PMC10687132 37603200

[92] Piri Damaghi M Mirzababaei A Moradi S Daneshzad E Tavakoli A Clark CCT . Comparison of the effect of soya protein and whey protein on body composition: a meta-analysis of randomised clinical trials. Br J Nutr. (2022) 127:885–95. doi: 10.1017/S0007114521001550 33971994

[93] Jenkins DJA Blanco Mejia S Chiavaroli L Viguiliouk E Li SS Kendall CWC Vuksan V . Cumulative meta-analysis of the soy effect over time. J Am Heart Assoc. (2019) 8:e012458. doi: 10.1161/JAHA.119.012458 PMC6662359 31242779

[94] Yimam MA Roberts J O'Callaghan A Holwerda A Cassidy Y Luiking Y . Long-term effects of plant vs. animal protein supplementation on body composition, muscle strength, physical performance, and cardiometabolic risk factors in adults: a systematic review and meta-analysis of randomized controlled trials. Front Nutr. (2026) 13:1813846. doi: 10.3389/fnut.2026.1813846 PMC13078973 41994089

[95] Lichtenstein AH Khera A Anderson CAM Appel LJ DeSilva DM Gardner C . Dietary guidance to improve cardiovascular health: a scientific statement from the American Heart Association. Circulation (2026) 153. doi: 10.1161/CIR.0000000000001435 41914202

[96] Zhou S Cheng F He J Xu T Zhang X Wan S . Effects of high-quality protein supplementation on cardiovascular risk factors in individuals with metabolic diseases: a systematic review and meta-analysis of randomized controlled trials. Clin Nutr (2024) 43:1740–50. doi: 10.1016/j.clnu.2024.06.013 38924998

[97] Joy JM Lowery RP Wilson JM Purpura M De Souza EO Wilson SM . The effects of 8 weeks of whey or rice protein supplementation on body composition and exercise performance. Nutr J. (2013) 12:86. doi: 10.1186/1475-2891-12-86 PMC3698202 23782948

[98] Babault N Païzis C Deley G Guérin-Deremaux L Saniez MH Lefranc-Millot C . Pea proteins oral sup-plementation promotes muscle thickness gains during resistance training: a double-blind, randomized, placebo-controlled clinical trial vs. whey protein. J Int Soc Sports Nutr. (2015) 12:3. doi: 10.1186/s12970-014-0064-5 PMC4307635 25628520

[99] Govindasamy K Parpa K Katanic B Clark CCT Elayaraja M Kambitta Valappil IN . Effect of plant-based proteins on recovery from resistance exercise-induced muscle damage in healthy young adults: a systematic review. Nutrients (2025) 17:2571. doi: 10.3390/nu17152571 PMC12348865 40806155

[100] Zhao S Zhang H Xu Y Li J Du S Ning Z. The effect of protein intake on athletic performance: a systematic review and meta-analysis. Front Nutr. (2024) 11:1455728. doi: 10.3389/fnut.2024.1455728 PMC11613885 39628467

[101] Lonnie M Laurie I Myers M Horgan G Russell WR Johnstone AM. Exploring health-promoting attributes of plant proteins as a functional ingredient for the food sector: a systematic review of human interventional studies. Nutrients. (2020) 12:2291. doi: 10.3390/nu12082291 PMC7468935 32751677

[102] Sarwar Gilani G Wu Xiao C Cockell KA. Impact of antinutritional factors in food proteins on the digestibility of protein and the bioavailability of amino acids and on protein quality. Br J Nutr. (2012) 108:S315–32. doi: 10.1017/S0007114512002371 23107545

[103] Crozier SJ Kimball SR Emmert SW Anthony JC Jefferson LS. Oral leucine administration stimulates protein synthesis in rat skeletal muscle. J Nut. (2005) 135:376–82. doi: 10.1093/jn/135.3.376 15735066

[104] Wolfson RL Chantranupong L Saxton RA Shen K Scaria SM Cantor JR . Sestrin2 is a leucine sensor for the mTORC1 pathway. Science (2016) 351:43–8. doi: 10.1126/science.aab2674 PMC4698017 26449471

[105] Saxton RA Sabatini DM. mTOR signaling in growth, metabolism, and disease. Cell. (2017) 168:960–76. doi: 10.1016/j.cell.2017.02.004 PMC5394987 28283069

[106] Wolfson RL Sabatini DM. The dawn of the age of amino acid sensors for the mTORC1 pathway. Cell Metab (2017) 26, 301–9. doi: 10.1016/j.cmet.2017.07.001 PMC5560103 28768171

[107] Lama-Sherpa TD Jeong MH Jewell JL. Regulation of mTORC1 by the rag GTPases. Biochem Soc Trans. (2023) 51:655–64. doi: 10.1042/BST20210038 PMC10212514 36929165

[108] Pennings B Groen B de Lange A Gijsen AP Zorenc AH Senden JM . Amino acid absorption and subsequent muscle protein accretion following graded intakes of whey protein in elderly men. Am J Physiol Endocrinol Metab. (2012) 302:E992–9. doi: 10.1152/ajpendo.00517.2011 22338070

[109] Mitchell CJ Churchward-Venne TA Parise G Bellamy L Baker SK Smith K . Acute post-exercise myofibrillar protein synthesis is not correlated with resistance training-induced muscle hypertrophy in young men. PLoS ONE. (2014) 9:e89431. doi: 10.1371/journal.pone.0089431 PMC3933567 24586775

[110] Phillips SM. Current concepts and unresolved questions in dietary protein requirements and supplements in adults. Front Nutr. (2017) 4:13. doi: 10.3389/fnut.2017.00013 PMC5420553 28534027

[111] Jakubowski JS Nunes EA Teixeira FJ Vescio V Morton RW Banfield L . Supplementation with the leucine metabolite β-hydroxy-β-methylbutyrate (HMB) does not improve resistance exercise-induced changes in body composition or strength in young subjects: a systematic review and meta-analysis. Nutrients. (2020) 12:1523. doi: 10.3390/nu12051523 PMC7285233 32456217

[112] Atherton PJ Smith K. Muscle protein synthesis in response to nutrition and exercise. J Physiol. (2012) 590:1049–57. doi: 10.1113/jphysiol.2011.225003 PMC3381813 22289911

[113] Shad BJ Thompson JL Breen L. Does the muscle protein synthetic response to exercise and amino acid-based nutrition diminish with advancing age? A systematic review. Am J Physiol Endocrinol Metab. (2016) 311:E803–17. doi: 10.1152/ajpendo.00213.2016 27555299

[114] Guo Y Fu X Hu Q Chen L Zuo H. The effect of leucine supplementation on sarcopenia-related measures in older adults: a systematic review and meta-analysis of 17 randomized controlled trials. Front Nutr. (2022) 9:929891. doi: 10.3389/fnut.2022.929891 PMC9284268 35845777

[115] Eley HL Russell ST Tisdale MJ. Mechanism of attenuation of muscle protein degradation induced by tumor necrosis factor-α and angiotensin II by β-hydroxy-β-methylbutyrate. Am J Physiol Endocrinol Metab. (2008) 295:E1417–26. doi: 10.1152/ajpendo.90567.2008 18840762

[116] Wilkinson DJ Hossain T Hill DS Phillips BE Crossland H Williams J . Effects of leucine and its metabolite β-hydroxy-β-methylbutyrate on human skeletal muscle protein metabolism. J Physiol. (2013) 591:2911–23. doi: 10.1113/jphysiol.2013.253203 PMC3690694 23551944

[117] Holeček M. β-hydroxy-β-methylbutyrate supplementation and skeletal muscle in healthy and muscle-wasting conditions. J Cachexia Sarcopenia Muscle. (2017) 8:529–41. doi: 10.1002/jcsm.12208 PMC5566641 28493406

[118] Gu WT Zhang LW Wu FH Wang S. The effects of β-hydroxy-β-methylbutyrate supplementation in patients with sarcopenia: a systematic review and meta-analysis. Maturitas. (2025) 195:108219. doi: 10.1016/j.maturitas.2025.108219 39999663

[119] García-Alonso A Sánchez-González JL Navarro-López V Méndez-Sánchez R Polo-Ferrero L. The role of HMB supplementation in enhancing the effects of resistance training in older adults: a systematic review and meta-analysis on muscle quality, body composition, and physical function. Nutrients. (2025) 17:3624. doi: 10.3390/nu17223624 PMC12655442 41305674

[120] Hu YG Shi JH Yu DX Huang HB. The effects of beta-hydroxy-beta-methyl butyrate supplementation in surgical patients: a systematic review and meta-analysis of randomized controlled trials. Front Nutr. (2025) 12:1621206. doi: 10.3389/fnut.2025.1621206 PMC12326741 40771218

[121] D'Antona G Ragni M Cardile A Tedesco L Dossena M Bruttini F . Branched-chain amino acid supplementation promotes survival and supports cardiac and skeletal muscle mitochondrial biogenesis in middle-aged mice. Cell Metab. (2010) 12:362–372. doi: 10.1016/j.cmet.2010.08.016 20889128

[122] Moberg M Apró W Ekblom B van Hall G Holmberg HC Blomstrand E. Activation of mTORC1 by leucine is potentiated by branched-chain amino acids and even more so by essential amino acids following resistance exercise. Am J Physiol Cell Physiol. (2016) 310:C874–84. doi: 10.1152/ajpcell.00374.2015 27053525

[123] Wolfe RR. Branched-chain amino acids and muscle protein synthesis in humans: myth or reality? J Int Soc Sports Nutr. (2017) 14:30. doi: 10.1186/s12970-017-0184-9 PMC5568273 28852372

[124] Salem A Ben Maaoui K Jahrami H AlMarzooqi MA Boukhris O Messai B . Attenuating muscle damage biomarkers and muscle soreness after an exercise-induced muscle damage with branched-chain amino acid (BCAA) supplementation: a systematic review and me-ta-analysis with meta-regression. Sports Med Open. (2024) 10:42. doi: 10.1186/s40798-024-00686-9 PMC11021390 38625669

[125] Bai GH Tsai MC Tsai HW Chang CC Hou WH. Effects of branched-chain amino acid-rich supplementation on EWGSOP2 criteria for sarcopenia in older adults: a systematic review and meta-analysis. Eur J Nutr. (2022) 61:637–51. doi: 10.1007/s00394-021-02710-0 34705076

[126] Volpi E Kobayashi H Sheffield-Moore M Mittendorfer B Wolfe RR. Essential amino acids are primarily responsible for the amino acid stimulation of muscle protein anabolism in healthy elderly adults. Am J Clin Nutr. (2003) 78:250–8. doi: 10.1093/ajcn/78.2.250 PMC3192452 12885705

[127] Paddon-Jones D Sheffield-Moore M Katsanos CS Zhang XJ Wolfe RR. Differential stimulation of muscle protein synthesis in elderly humans following ingestion of essential amino acids. Am J Physiol Endocrinol Metab. (2004) 286:E321–8. doi: 10.1152/ajpendo.00368.2003 14583440

[128] Rondanelli M Aquilani R Verri M Boschi F Pasini E Perna S . Plasma kinetics of essential amino acids following their ingestion as free formula or as dietary protein components. Aging Clin Exp Res. (2017) 29:801–5. doi: 10.1007/s40520-016-0605-7 27406393

[129] Aquilani R Viglio S Iadarola P Opasich C Testa A Dioguardi FS . Oral amino acid supplements improve exercise capacities in elderly patients with chronic heart failure. Am J Cardiol. (2008) 101:104E−10E. doi: 10.1016/j.amjcard.2008.03.008 18514618

[130] Aquilani R D'Antona G Baiardi P Gambino A Iadarola P Viglio S . Essential amino acids and exercise tolerance in elderly muscle-depleted subjects with chronic diseases: a rehabilitation without reha-bilitation? Biomed Res Int. (2014) 2014:341603. doi: 10.1155/2014/341603 PMC4070286 25009815

[131] Aquilani R Zuccarelli Ginetto C Rutili C Pisano P Pasini E Baldissarro E . Supplemented amino acids may enhance the walking recovery of elderly subjects after hip fracture surgery. Aging Clin Exp Res. (2019) 31:157–60. doi: 10.1007/s40520-018-0941-x 29667153

[132] Volpato S Custureri R Puntoni M Bianchi L Daragjati J Garaboldi S . Effects of oral amino acid supplementation on multidimensional prognostic index in hospitalized older patients: a multicenter randomized, double-blind, placebo-controlled pilot study. Clin Interv Aging. (2018) 13, 633–40. doi: 10.2147/CIA.S154998 PMC5908340 29713147

[133] Negro M Crisafulli O D'Antona G. Effects of essential amino acid (EAA) and glutamine supplementation on skeletal muscle wasting in acute, subacute, and postacute conditions. Clin Nutr ESPEN. (2024) 62:224–33. doi: 10.1016/j.clnesp.2024.05.023 38843393

[134] Ferrando AA Wolfe RR Hirsch KR Church DD Kviatkovsky SA Roberts MD . International Society of Sports Nutrition position stand: effects of essential amino acid supplementation on exercise and performance. J Int Soc Sports Nutr. (2023) 20:2263409. doi: 10.1080/15502783.2023.2263409 PMC10561576 37800468

[135] Lombardi C Carubelli V Lazzarini V Vizzardi E Quinzani F Guidetti F . Effects of oral amino acid supplements on functional capacity in patients with chronic heart failure. Clin Med Insights Cardiol. (2014) 8:39–44. doi: 10.4137/CMC.S14016 PMC4039212 24899826

[136] Hagiyama A Yamamoto N Watanabe J Tsuge T Nakashima Y Nakao S . Efficacy of protein and essential amino acid supplementation in lower limb surgeries: a systematic review and meta-analysis. Cureus. (2024) 16:e69212. doi: 10.7759/cureus.69212 PMC11469702 39398772

[137] Brown E Mohler SA Kviatkovsky SA Blake LE Hill JR Stambough JB . Amino Acid Supplementation May Help Prevent Muscle Wasting After Orthopedic Surgery, but Additional Studies Are Warranted: A Systematic Review of Randomized Clinical Trials. HSS J 2025, 21(2), 200–210. doi: 10.1177/15563316241308265 PMC11713956 39802328

[138] Armentaro G Cassano V Loiacono P Fuoco C Severini G Pastura CA . Early effect of supplementation with essential amino acids on cardiac performance in elderly patients with heart failure and sarcopenia. Int J Mol Sci. (2025) 26:7533. doi: 10.3390/ijms26157533 PMC12346981 40806660

[139] Fearon K Strasser F Anker SD Bosaeus I Bruera E Fainsinger RL . Definition and classification of cancer cachexia: an international consensus. Lancet Oncol. (2011) 12:489–95. doi: 10.1016/S1470-2045(10)70218-7 21296615

[140] Fearon K Arends J Baracos V. Understanding the mechanisms and treatment options in cancer cachexia. Nat Rev Clin Oncol. (2013) 10:90–9. doi: 10.1038/nrclinonc.2012.209 23207794

[141] Tisdale M. Cachexia in cancer patients. Nat Rev Cancer. (2002) 2:862–71. doi: 10.1038/nrc927 12415256

[142] Pradhan R Dieterich W Natarajan A Schwappacher R Reljic D Herrmann HJ . Influence of amino acids and exercise on muscle protein turnover, particularly in cancer cachexia. Cancers. (2024) 16:1921. doi: 10.3390/cancers16101921 PMC11119313 38791998

[143] Roeland EJ Bohlke K Baracos VE Bruera E Del Fabbro E Dixon S . Management of cancer cachexia: ASCO guideline. J Clin Oncol. (2020) 38:2438–53. doi: 10.1200/JCO.20.00611 32432946

[144] Braha A Albai A Timar B Negru Ş Sorin S Roman D . Nutritional interventions to improve cachexia outcomes in cancer: a systematic review. Medicina. (2022) 58:966. doi: 10.3390/medicina58070966 PMC9318456 35888685

[145] McCue MV MacLean DA. Amino acid metabolism in cancer cachexia and chemotherapy myotoxicity. Am J Physiol Cell Physiol. (2025) 329:C1451–67. doi: 10.1152/ajpcell.00085.2025 40929140

[146] Ligibel JA Bohlke K May AM Clinton SK Demark-Wahnefried W Gilchrist SC . Exercise, diet, and weight management during cancer treatment: ASCO guideline. J Clin Oncol. (2022) 40:2491–507. doi: 10.1200/JCO.22.00687 35576506

[147] Corsetti G Pasini E D'Antona G Nisoli E Flati V Assanelli D . Morphometric changes induced by amino acid supplementation in skeletal and cardiac muscles of old mice. Am J Cardiol. (2008) 101:26–34E. doi: 10.1016/j.amjcard.2008.02.078 18514623

[148] Corsetti G D'Antona G Ruocco C Stacchiotti A Romano C Tedesco L . Dietary supplementation with essential amino acids boosts the beneficial effects of rosuvastatin on mouse kidney. Amino Acids. (2014) 46:2189–203. doi: 10.1007/s00726-014-1772-5 PMC4133027 24923264

[149] Corsetti G Romano C Pasini E Marzetti E Calvani R Picca A . Diet enrichment with a specific essential free amino acid mixture improves healing of undressed wounds in aged rats. Exp Gerontol. (2017) 96:138–45. doi: 10.1016/j.exger.2017.06.020 28669821

[150] Lee KW Chung S Kim E Park YJ Ha K. Protein supplementation in South Korea: balancing physiological benefits and metabolic risks for evidence-based guidelines. J Nutr Health. (2026) 59:159–75. doi: 10.4163/jnh.2026.59.2.159

[151] Martin WF Armstrong LE Rodriguez NR. Dietary protein intake and renal function. Nutr Metab. (2005) 2:25. doi: 10.1186/1743-7075-2-25 PMC1262767 16174292

[152] Antonio J Ellerbroek A Silver T Vargas L Tamayo A Buehn R . A high protein diet has no harmful effects: A one-year crossover study in resistance-trained males. J Nutr Meta. (2016) 2016:9104792. doi: 10.1155/2016/9104792 PMC5078648 27807480

[153] Kidney Disease: improving Global Outcomes (KDIGO) CKD Work Group. KDIGO 2024 Clinical Practice Guideline for the Evaluation and Management of Chronic Kidney Disease. Kidney Int. (2024) 105:S117–314. 10.1016/j.kint.2023.10.018 38490803

[154] Kalantar-Zadeh K Fouque D. Nutritional management of chronic kidney disease. N Engl J Med. (2017) 377:1765–76. doi: 10.1056/NEJMra1700312 29091561

[155] Kalantar-Zadeh K Kramer HM Fouque D. High-protein diet is bad for kidney health: unleashing the taboo. Nephrol Dial Transplant. (2020) 35:1–4. doi: 10.1093/ndt/gfz216 31697325

[156] Giovannetti S Maggiore Q. A low-nitrogen diet with proteins of high biological value for severe chronic uraemia. Lancet. (1964) 283:1000–3. doi: 10.1016/S0140-6736(64)91919-1 14129799

[157] Lynch CJ Adams SH. Branched-chain amino acids in metabolic signalling and insulin resistance. Nat Rev Endocrinol. (2014) 10:723–36. doi: 10.1038/nrendo.2014.171 PMC4424797 25287287

[158] White PJ Newgard CB. Branched-chain amino acids in disease. Science. (2019) 363:582–3. doi: 10.1126/science.aav0558 PMC9940269 30733403

[159] Maughan RJ. Quality assurance issues in the use of dietary supplements, with special reference to protein supplements. J Nutr. (2013) 143:1843S−7S. doi: 10.3945/jn.113.176651 24027186

[160] Garthe I Maughan RJ. Athletes and supplements: prevalence and perspectives. Int J Sport Nutr Exerc Metab. (2018) 28:126–38. doi: 10.1123/ijsnem.2017-0429 29580114

[161] Bandara SB Towle KM Monnot AD. A human health risk assessment of heavy metal ingestion among consumers of protein powder supplements. Toxicol Rep. (2020) 7:1255–62. doi: 10.1016/j.toxrep.2020.08.001 PMC7509468 33005567

[162] Bethencourt-Barbuzano E de Lima Brum R Martín-León V Paz-Montelongo S Gutiérrez-Fernández ÁJ da Silva Júnior FMR . Quantifying lead and cadmium in protein supplements: Insights into dietary exposure and health risks. J Food Compos Anal. (2025) 148:108516. doi: 10.1016/j.jfca.2025.108516

[163] Thomas DT Erdman KA Burke LM. Position of the academy of nutrition and dietetics, dietitians of Canada, and the American college of sports medicine: nutrition and athletic performance. J Acad Nutr Diet. (2016) 116:501–28. doi: 10.1016/j.jand.2015.12.006 26920240

[164] Blachier F. Relevance and safe utilization of amino acids in supplements for human nutrition: lessons from clinical and pre-clinical studies. Nutrients. (2026) 18:296. doi: 10.3390/nu18020296 PMC12845092 41599909

[165] Tu DY Kao FM Tsai ST Tung TH. Sarcopenia among the elderly population: a systematic review and meta-analysis of randomized controlled trials. Healthcare. (2021) 9:650. doi: 10.3390/healthcare9060650 PMC8228648 34072617

[166] Björkman MP Suominen MH Kautiainen H Jyväkorpi SK Finne-Soveri HU Strandberg TE et al. Effect of protein supplementation on physical performance in older people with sarcopenia: a randomized controlled trial. J Am Med Dir Assoc (2020) 21:226–32.e1. doi: 10.1016/j.jamda.2019.09.006 31734121

[167] Weijs PJ Cynober L DeLegge M Kreymann G Wernerman J Wolfe RR . Proteins and amino acids are fundamental to optimal nutrition support in critically ill patients. Crit Care. (2014) 18:591. doi: 10.1186/s13054-014-0591-0 PMC4520087 25565377

[168] Vieillevoye S Poortmans JR Duchateau J Carpentier A. Effects of a combined essential amino acids/carbohydrate sup-plementation on muscle mass, architecture and maximal strength following heavy-load training. Eur J Appl Physiol. (2010) 110:479–88. doi: 10.1007/s00421-010-1520-9 20521064

